# Parkinson's Disease: The Epidemiology, Risk Factors, Molecular Pathogenesis, Prevention, and Therapy

**DOI:** 10.1002/mco2.70540

**Published:** 2025-12-12

**Authors:** Xue‐Yao Guo, Dong‐Yan Song, Ming‐Yang Wu, Jing‐Qi Zhang, Jia‐Yi Li, Lin Yuan

**Affiliations:** ^1^ Shengjing Hospital China Medical University Shenyang China; ^2^ Health Sciences Institute Key Laboratory of Major Chronic Diseases of Nervous System of Liaoning Province China Medical University Shenyang China; ^3^ Neural Plasticity and Repair Unit Department of Experimental Medical Sciences Lund University Lund Sweden

**Keywords:** α‑synuclein, blood transfusion, gut–brain axis, protein aggregation, Parkinson's disease

## Abstract

Parkinson's disease (PD) is a progressive neurodegenerative disorder with a growing global burden. Current pharmacological therapies remain limited to symptomatic management, owning to an incomplete understanding of the mechanisms driving α‑synuclein aggregation and disease progression. This review provides an integrated overview of PD across epidemiological, etiological, pathophysiological, and clinical dimensions. It emphasizes established and emerging risk factors, including environmental toxins, lifestyle variables, and gut microbiota dysbiosis and delineates how peripheral–central pathways such as the gut–brain, erythrocyte–brain, and kidney–brain axes contribute to PD pathogenesis. At the molecular level, we explore key disruptions including proteostatic failure, aberrant phase separation, oxidative stress, neuroinflammation, synaptic dysfunction, iron dyshomeostasis, and impaired cholesterol metabolism. These encompass microbiome‑targeted interventions and blood‐based approaches. We further evaluate a spectrum of management strategies ranging from primary prevention and biomarker‑guided early detection to innovative experimental treatments such as cellular therapies, transfusion‑based modalities, and microbial modulation. By integrating recent advances in systemic pathophysiology with translational perspectives, this review highlights how molecular and cellular dysregulations underlie clinical phenotypes. Finally, we discuss promising biomarkers derived from microbial, inflammatory, and erythrocyte pathways that may facilitate early diagnosis and the development of disease‑modifying therapies.

## Graphical abstract

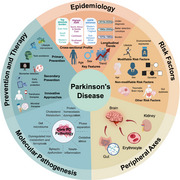

## 1 Introduction

Parkinson's disease (PD) is a progressive neurodegenerative disorder that predominantly affects the elderly population. Globally, it is estimated to affect more than 10 million individuals, with the majority of cases emerging after the age of 60 years. Pathologically, PD is characterized by degeneration of dopaminergic neurons in the substantia nigra pars compacta (SNpc) and accumulation of misfolded α‐synuclein (α‑syn), which aggregates into intracellular inclusions known as Lewy bodies (LBs) and Lewy neurites [1, 2]. These pathological changes underlie the characteristic clinical features of PD. Clinically, PD presents with cardinal motor symptoms including bradykinesia, resting tremor, muscular rigidity, and postural instability [3]. Additionally, a broad spectrum of nonmotor symptoms is commonly observed, such as sleep disturbances, constipation, depression, olfactory dysfunction, and cognitive impairment [4, 5, 6, 7]. Many of these nonmotor features may precede overt motor dysfunction by several years, suggesting their potential role as early indicators of disease onset [3, 8].

The pathogenesis of PD involves several interconnected processes centered on α‑syn aggregation, dopaminergic neurodegeneration, and neuroinflammation. Misfolded α‑syn, a presynaptic neuronal protein, forms soluble oligomers and insoluble fibrils that overwhelm cellular proteostasis mechanisms, leading to progressive intraneuronal accumulation [9, 10]. These toxic α‑syn species disrupt membrane integrity, impair mitochondrial and lysosomal function, and trigger neuroinflammatory responses [11]. Concomitantly, selective degeneration of nigrostriatal dopaminergic neurons results in severe dopamine depletion within the basal ganglia circuitry [12, 13]. Neuroinflammation further amplifies these degenerative processes through microglial activation and sustained release of proinflammatory cytokines, creating a vicious cycle that exacerbates neuronal loss [14, 15, 16, 17]. These cumulative pathological changes contribute to the principal clinical manifestations of PD: (1) the classic motor symptoms (resting tremor, bradykinesia, rigidity, and postural instability) are primarily associated with dopaminergic circuit degeneration [6, 18]; and (2) the diverse nonmotor symptoms (sleep disturbances, olfactory dysfunction, mood disorders, and cognitive decline) are linked to widespread α‑syn pathology in limbic and cortical regions [19, 20, 21, 22, 23]. Chronic neuroinflammation and α‑syn‑mediated cellular stress further contribute to behavioral and cognitive impairments, underscoring the multifaceted and system‑wide nature of PD pathophysiology [13, 14, 15, 16, 17, 24].

Emerging evidence implicates peripheral–central crosstalk, with the gastrointestinal tract posited as a potential initial site of α‑syn aggregation, possibly triggered by enteric infections, or local immune activation [19, 20, 25, 26]. Pathogenic forms of α‐syn are propagate to the central nervous system (CNS) via retrograde transport along the vagus nerve, supporting mechanistic support for the gut–brain axis hypothesis in PD [21, 23, 27]. In addition to neural pathways, hematogenous routes may facilitate the transmission of pathology. Circulating α‑syn and proinflammatory mediators are thought to compromise blood–brain barrier (BBB) integrity and gain access to the CNS, suggesting the role of the erythrocyte–brain axis in the peripheral‑to‑central propagation of pathology [28, 29, 30, 31]. Furthermore, recent studies identify the involvement of a kidney–brain axis, wherein renal dysfunction may exacerbate neurodegeneration through impaired clearance of neurotoxic metabolites, chronic systemic inflammation, and metabolic dysregulation [32, 33, 34]. Collectively, these multidirectional interactions illustrate that PD is not confined to the brain but reflects a broader system‑level dysregulation.

This review provides an integrative overview of PD by examining the interrelationships among epidemiological patterns, risk factors, pathophysiological mechanisms, and therapeutic strategies. Rather than addressing these domains in isolation, it highlights how progress in one area informs understanding in others. We first construct multidimensional framework for PD that incorporates: (1) population‑level evidence including disease prevalence, temporal trends, and risk exposures; (2) systemic mechanisms including gut–brain, erythrocyte–brain, and kidney–brain axes; and (3) molecular and cellular dysregulations involving α‑syn aggregation, mitochondrial impairment, oxidative stress, neuroinflammation, and iron dyshomeostasis. Presenting these elements within a unified structure underscores the links between external influences and intrinsic disease processes. Based on this investigative framework, the review subsequently evaluates current and emerging therapeutic strategies with their biological foundations. Preventions are discussed within the context of established risk factors and modifiable behaviors, while biomarker‑based early detection bridges to timely interventions. Novel mechanism‑informed therapeutic approaches, such as blood‑based and microbiome‑targeted interventions are examined, reflecting a growing focus on systemic disease modulation. Finally, the review addresses the translation of mechanism‐based therapeutic strategies into clinical practice. It highlights recurring challenges across interventions, such as efficacy validation, implementation feasibility, and patient‑specific variability, while emphasizing the need to align biological insights with practical delivery. Collectively, these discussions ultimately aim to reduce the global burden of PD and improve the quality of life for affected individuals.

## 2 Epidemiology

PD is a progressive neurodegenerative disorder that causes prolonged disability in late life and imposes a growing burden on healthcare systems worldwide. A thorough epidemiological understanding is therefore essential for understanding its evolving impact and guiding effective public health strategies. This section summarizes the current cross‑sectional profile of PD stratified by age‑, sex‑, and region‑, reviews temporal trends over the past five decades, and discusses persistent knowledge gaps and future research priorities, with particular attention to underserved and underrepresented populations.

### 2.1 Cross‑Sectional Epidemiological Profile

According to the Global Burden of Disease, Injuries, and Risk Factors Study (GBD), PD affected an estimated 11.67 million individuals globally and was associated with approximately 0.42 million deaths in 2023. The age‑standardized prevalence rate (ASPR) was 128.5 per 100,000 population, with an age‑standardized incidence rate (ASIR) of 15.11 per 100,000, and age‑standardized disability‑adjusted life years (DALYs) of 88.62 per 100,000 person‑years [35]. Besides, the World Health Organization (WHO) reported over 8.5 million PD cases in 2019—nearly double the number documented in 1996 [36]. Notably, PD has exhibited the faster growth among neurological disorders in terms of both DALYs and mortality over recent decades [37].

Significant geographic disparities exist in the global distribution of PD. In 2023, East Asia, South Asia, and Western Europe accounted for the highest absolute numbers of incident cases, prevalent cases, and DALYs [35]. Conversely, North Africa and the Middle East, Caribbean, and Southeast Asia exhibited the highest ASIR, indicating particularly high disease burden in North Africa and the Middle East. Western Europe demonstrated the highest ASPR at approximately 1.6‰, in sharp contrast to Sub‑Saharan Africa, which had the lowest recorded ASPR of around 0.05‰ [38] (see Table 1). These differences likely reflect variations in population aging, healthcare access, and diagnostic capacities across regions. Further supporting global heterogeneity, a 2024 meta‑analysis published in *Nature* reported PD prevalence estimates ranging from 49 per 100,000 in Sub‑Saharan Africa to 1081 per 100,000 in Latin America [39], figures that suggest even greater regional variability than captured in GBD estimates. Overall, high‑ and middle‑sociodemographic index (SDI) regions were associated with elevated ASIRs, whereas low‑SDI regions experienced the most rapid incidence growth, potentially reflecting uneven case ascertainment and demographic variations.

**TABLE 1:** Global age‑standardized prevalence and sex differences in age‑standardized prevalence of Parkinson's disease by country in 2023.

Location name	Total prevalence (per 100,000)	(95% UI)	Male–female difference (per 100,000)	95% UI
China
The Chinese mainland	148.7	(122.1–183.5)	25.8	(21.8–33.6)
Taiwan (Province of China)	239.9	(232.6–248.1)	51.1	(51.0–51.4)
Democratic People's Republic of Korea	133.8	(111.3–162.4)	35.8	(34.0–42.5)
Cambodia	92.0	(77.6–110.9)	34.7	(28.2–48.0)
Indonesia	88.0	(72.7–108.6)	35.1	(28.3–44.3)
Lao People's Democratic Republic	80.5	(67.3–96.4)	31.1	(24.9–41.1)
Malaysia	100.9	(83.9–120.5)	39.4	(33.1–49.9)
Maldives	111.0	(93.7–130.3)	36.0	(33.4–41.8)
Myanmar	82.6	(67.0–100.5)	28.1	(25.8–32.8)
Philippines	82.5	(67.9–102.1)	41.3	(33.5–51.6)
Sri Lanka	113.3	(96.2–131.7)	46.5	(37.6–46.0)
Thailand	108.9	(90.3–127.8)	40.9	(33.9–51.0)
Timor‐Leste	88.8	(74.7–104.7)	25.2	(21.7–28.5)
Viet Nam	101.2	(83.3–122.6)	31.7	(25.3–46.5)
Fiji	141.2	(119.3–169.2)	50.6	(41.6–53.9)
Kiribati	147.5	(124.9–177.9)	28.0	(25.4–36.1)
Marshall Islands	164.6	(137.0–199.2)	36.3	(27.6–37.8)
Micronesia (Federated States of)	158.3	(131.6–186.8)	48.2	(44.2–58.2)
Papua New Guinea	146.5	(120.9–177.8)	7.4	(8.3–2.5)
Samoa	145.0	(119.9–170.0)	22.7	(16.2–27.0)
Solomon Islands	120.1	(99.3–145.7)	32.6	(26.2–38.0)
Tonga	143.7	(122.2–172.4)	40.3	(34.9–46.9)
Vanuatu	121.9	(103.5–142.4)	33.3	(25.0–44.6)
Armenia	36.2	(30.3–42.4)	12.1	(10.5–14.2)
Azerbaijan	34.3	(30.2–38.9)	13.4	(11.6–14.7)
Georgia	42.5	(35.7–51.9)	15.8	(12.2–17.1)
Kazakhstan	41.9	(34.8–51.0)	16.8	(13.9–18.5)
Kyrgyzstan	32.2	(27.2–39.6)	14.3	(12.3–15.9)
Mongolia	27.8	(24.8–31.1)	5.2	(4.3–5.9)
Tajikistan	47.6	(39.6–56.4)	12.8	(10.2–16.2)
Turkmenistan	36.1	(30.5–43.5)	14.9	(12.4–19.1)
Uzbekistan	35.0	(29.2–42.3)	13.8	(10.3–17.1)
Albania	111.3	(91.4–134.9)	30.4	(22.1–43.1)
Bosnia and Herzegovina	107.7	(87.3–128.4)	44.2	(33.5–63.9)
Bulgaria	109.1	(90.6–124.8)	22.3	(25.7–19.5)
Croatia	110.2	(91.9–132.8)	22.1	(19.1–25.6)
Czechia	112.0	(92.9–125.9)	26.8	(32.6–24.4)
Hungary	122.1	(116.2–129.3)	93.5	(90.1–97.6)
North Macedonia	113.2	(91.0–134.2)	39.1	(29.5–46.7)
Montenegro	109.8	(88.8–132.4)	39.0	(24.5–53.1)
Poland	112.9	(100.9–125.7)	38.7	(34.4–43.2)
Romania	104.5	(86.9–118.9)	42.6	(42.0–47.7)
Serbia	100.1	(83.6–116.4)	37.4	(31.1–43.8)
Slovakia	94.8	(77.5–114.3)	39.4	(26.9–52.3)
Slovenia	113.0	(93.4–128.4)	46.9	(42.7–45.8)
Belarus	52.4	(43.2–62.6)	29.4	(22.0–34.1)
Estonia	107.7	(98.4–118.3)	51.9	(51.1–52.9)
Latvia	61.5	(51.8–69.5)	36.7	(28.8–39.8)
Lithuania	59.7	(49.6–69.5)	31.7	(27.4–41.1)
Republic of Moldova	49.4	(39.2–58.5)	31.6	(24.7–36.4)
Russian Federation	57.2	(45.9–69.1)	29.3	(23.7–34.6)
Ukraine	26.6	(22.0–32.1)	26.5	(21.4–32.7)
Brunei Darussalam	87.3	(70.9–106.2)	52.9	(43.9–66.3)
Japan	88.2	(71.7–108.1)	30.9	(24.7–39.7)
Republic of Korea	119.4	(112.9–128.3)	5.0	(4.2–4.8)
Singapore	61.1	(51.0–72.7)	35.9	(30.1–45.8)
Australia	154.9	(127.0–185.2)	79.5	(61.2–87.3)
New Zealand	108.2	(87.4–134.8)	45.3	(35.8–57.6)
Andorra	96.7	(80.9–115.8)	42.9	(35.5–53.9)
Austria	109.2	(95.5–125.9)	45.4	(50.3–39.0)
Belgium	109.0	(94.9–128.8)	41.0	(38.0–48.0)
Cyprus	113.5	(94.4–132.5)	53.3	(44.8–62.8)
Denmark	95.5	(86.6–108.1)	70.2	(64.9–78.8)
Finland	110.6	(97.9–123.6)	54.2	(51.6–55.6)
France	116.6	(108.7–124.2)	44.6	(41.2–50.0)
Germany	197.8	(187.3–209.7)	72.3	(71.7–73.3)
Greece	136.0	(119.9–152.5)	69.1	(55.9–78.4)
Iceland	143.8	(121.0–180.3)	59.0	(48.6–83.6)
Ireland	109.4	(94.5–125.3)	46.3	(41.0–47.9)
Israel	154.0	(135.3–184.4)	106.8	(90.8–129.2)
Italy	215.7	(178.5–263.4)	69.6	(56.6–87.1)
Luxembourg	118.1	(99.0–135.4)	55.9	(49.1–61.3)
Malta	121.0	(100.8–144.0)	61.6	(45.3–74.4)
Netherlands	111.2	(99.7–128.2)	53.8	(49.6–57.2)
Norway	114.7	(94.6–140.9)	44.7	(36.3–58.0)
Portugal	123.6	(109.4–141.4)	72.9	(63.9–79.0)
Spain	169.6	(143.9–195.1)	81.7	(74.8–87.9)
Sweden	163.9	(146.1–182.9)	64.9	(51.8–75.4)
Switzerland	117.1	(98.7–132.2)	47.4	(38.9–50.4)
United Kingdom	116.4	(97.2–139.1)	40.9	(34.4–48.4)
Argentina	144.9	(129.3–159.5)	279.2	(247.1–305.4)
Chile	126.5	(111.1–152.1)	274.1	(241.7–332.1)
Uruguay	156.0	(136.4–171.2)	336.3	(291.8–369.6)
Canada	118.0	(101.8–138.8)	49.7	(55.1–61.2)
United States of America	154.6	(137.7–173.1)	90.0	(80.2–97.9)
Antigua and Barbuda	172.6	(143.0–204.6)	79.3	(58.6–90.8)
Bahamas	179.1	(152.1–210.7)	67.4	(55.7–80.9)
Barbados	189.1	(155.6–226.8)	50.9	(42.9–56.4)
Belize	190.5	(158.3–226.5)	56.7	(42.7–74.1)
Cuba	154.8	(127.9–185.2)	45.6	(40.7–53.2)
Dominica	204.9	(168.6–237.7)	64.5	(55.9–75.4)
Dominican Republic	203.0	(168.4–238.5)	101.6	(84.2–125.0)
Grenada	193.3	(163.4–228.2)	73.5	(46.9–106.9)
Guyana	161.5	(133.9–194.4)	45.1	(36.8–57.0)
Haiti	136.9	(112.7–162.8)	37.4	(31.4–44.2)
Jamaica	152.9	(123.9–185.7)	44.1	(43.7–65.0)
Saint Lucia	190.6	(161.6–224.8)	78.8	(67.5–97.6)
Saint Vincent and the Grenadines	179.2	(142.9–211.2)	66.8	(56.4–70.1)
Suriname	182.2	(153.8–214.4)	69.8	(55.5–96.6)
Trinidad and Tobago	185.0	(152.5–230.3)	62.7	(44.8–73.6)
Bolivia (Plurinational State of)	234.3	(196.4–276.4)	77.1	(68.8–85.2)
Ecuador	222.8	(187.0–263.0)	57.7	(40.3–84.1)
Peru	160.7	(130.6–190.3)	−5.0	(−4.2—7.8)
Colombia	119.0	(111.4–126.2)	47.1	(43.3–49.9)
Costa Rica	169.5	(141.3–202.1)	22.7	(14.9–34.8)
El Salvador	167.7	(137.1–202.8)	26.6	(27.1–28.7)
Guatemala	172.5	(136.6–208.9)	23.7	(25.8–26.3)
Honduras	181.3	(150.8–212.4)	16.8	(13.7–12.4)
Mexico	182.5	(148.9–223.3)	−2.8	(−0.4—6.3)
Nicaragua	161.5	(134.3–193.8)	31.2	(27.8–37.4)
Panama	171.8	(135.7–200.7)	25.8	(18.8–20.8)
Venezuela (Bolivarian Republic of)	157.7	(124.9–189.7)	39.8	(38.0–50.6)
Brazil	284.0	(229.0–346.1)	104.8	(85.7–125.4)
Paraguay	291.7	(237.0–344.6)	137.6	(104.8–161.4)
Algeria	158.4	(128.1–192.6)	76.1	(56.5–89.6)
Bahrain	179.9	(147.1–215.3)	97.3	(63.2–115.7)
Egypt	183.7	(148.2–224.5)	81.2	(68.1–101.9)
Iran (Islamic Republic of)	161.1	(133.1–196.6)	98.6	(81.2–119.3)
Iraq	197.5	(165.3–236.7)	121.7	(98.8–144.6)
Jordan	125.1	(102.6–153.2)	62.8	(50.9–74.5)
Kuwait	181.1	(152.2–215.4)	119.4	(99.6–136.8)
Lebanon	147.0	(123.9–176.0)	77.6	(61.8–94.5)
Libya	162.0	(133.2–198.7)	92.5	(74.7–111.4)
Morocco	150.1	(123.6–180.3)	82.0	(69.6–96.8)
Palestine	153.9	(130.4–180.5)	87.4	(71.0–99.6)
Oman	215.6	(176.3–253.2)	163.2	(135.5–195.1)
Qatar	177.4	(144.5–215.6)	86.1	(64.9–100.4)
Saudi Arabia	256.0	(203.8–307.4)	200.4	(156.0–251.6)
Syrian Arab Republic	169.4	(139.1–200.4)	100.5	(82.6–118.0)
Tunisia	148.7	(120.3–178.1)	94.8	(75.7–112.8)
Türkiye	341.7	(293.8–396.5)	212.4	(184.5–251.8)
United Arab Emirates	131.4	(107.6–158.2)	37.5	(25.8–44.7)
Yemen	115.2	(96.0–135.5)	59.9	(47.4–69.0)
Afghanistan	138.2	(117.7–168.3)	46.3	(37.6–57.3)
Bangladesh	82.8	(66.8–101.6)	9.6	(6.2–11.9)
Bhutan	82.5	(67.5–97.4)	13.3	(11.0–18.2)
India	67.4	(55.1–82.7)	9.5	(7.1–10.7)
Nepal	74.4	(61.6–87.8)	13.8	(11.2–14.3)
Pakistan	85.8	(70.5–104.8)	15.8	(14.2–19.3)
Angola	52.3	(43.0–62.6)	20.2	(16.1–24.5)
Central African Republic	43.1	(35.9–52.1)	18.7	(15.8–22.8)
Congo	53.4	(44.5–65.9)	20.1	(17.1–24.6)
Democratic Republic of the Congo	44.5	(36.3–52.8)	16.4	(13.0–22.1)
Equatorial Guinea	55.1	(45.3–65.4)	26.5	(22.4–31.4)
Gabon	62.9	(52.4–74.4)	34.0	(28.2–37.9)
Burundi	46.7	(37.6–56.7)	22.9	(17.0–27.4)
Comoros	64.2	(55.1–74.5)	26.7	(23.0–29.1)
Djibouti	55.4	(45.7–65.8)	24.1	(19.8–27.6)
Eritrea	52.6	(44.0–63.4)	23.9	(20.8–27.1)
Ethiopia	57.3	(46.3–70.7)	21.7	(18.0–26.0)
Kenya	37.3	(30.4–45.2)	23.2	(18.3–27.7)
Madagascar	39.1	(32.0–48.7)	17.2	(13.9–19.1)
Malawi	39.4	(32.2–46.9)	18.9	(14.5–26.3)
Mauritius	118.5	(100.4–135.3)	43.0	(38.9–49.4)
Mozambique	43.1	(35.3–51.7)	18.2	(14.1–23.2)
Rwanda	45.5	(36.7–55.3)	26.6	(22.3–33.8)
Seychelles	137.5	(118.9–164.6)	68.6	(62.0–75.8)
Somalia	41.2	(33.9–48.9)	13.1	(10.6–15.9)
United Republic of Tanzania	44.3	(36.4–52.6)	24.4	(20.4–29.4)
Uganda	45.0	(36.4–54.0)	27.1	(21.2–31.7)
Zambia	45.0	(35.6–54.7)	19.3	(14.6–25.0)
Botswana	51.8	(42.6–61.0)	22.8	(18.0–29.4)
Lesotho	44.1	(36.5–51.6)	19.1	(13.7–23.7)
Namibia	52.1	(43.2–62.1)	25.1	(20.1–29.1)
South Africa	54.4	(44.8–65.9)	32.0	(25.7–38.7)
Eswatini	51.1	(42.3–60.9)	29.8	(23.2–36.1)
Zimbabwe	48.9	(41.1–59.2)	41.6	(34.1–52.9)
Benin	54.4	(44.7–65.0)	18.7	(15.9–25.0)
Burkina Faso	47.1	(39.0–55.5)	12.6	(9.4–14.6)
Cameroon	57.8	(47.4–68.4)	26.3	(21.7–31.1)
Cabo Verde	69.2	(56.5–82.7)	40.2	(34.6–49.5)
Chad	43.1	(35.0–52.2)	13.5	(11.1–17.8)
Côte d'Ivoire	52.7	(43.6–62.6)	22.1	(18.1–27.5)
Gambia	50.9	(41.5–61.6)	17.7	(14.1–23.3)
Ghana	55.4	(46.3–66.6)	23.5	(18.8–31.3)
Guinea	44.4	(36.6–53.1)	14.2	(12.0–14.9)
Guinea‐Bissau	50.2	(41.7–59.5)	19.1	(15.6–21.2)
Liberia	53.9	(43.9–66.2)	16.6	(13.8–23.7)
Mali	52.3	(43.6–62.7)	14.3	(11.6–16.7)
Mauritania	53.2	(43.5–65.0)	12.2	(9.0–14.3)
Niger	44.0	(36.6–53.1)	12.1	(10.3–12.9)
Nigeria	51.4	(41.9–63.1)	18.7	(15.8–20.9)
Sao Tome and Principe	69.1	(56.9–82.6)	28.5	(24.8–37.9)
Senegal	55.6	(46.1–66.9)	23.5	(17.7–27.8)
Sierra Leone	49.4	(39.3–61.1)	18.2	(14.6–24.5)
Togo	45.9	(37.2–54.7)	17.9	(14.5–23.6)
American Samoa	168.6	(145.9–199.6)	53.7	(50.7–59.8)
Bermuda	166.7	(139.3–193.7)	61.7	(53.2–70.7)
Cook Islands	147.3	(120.0–176.6)	36.7	(29.8–46.7)
Greenland	100.1	(81.2–119.3)	58.3	(43.4–71.2)
Guam	139.5	(117.5–166.5)	49.5	(40.8–61.4)
Monaco	119.3	(96.7–140.2)	62.1	(47.8–73.0)
Nauru	160.2	(135.8–192.4)	49.7	(41.6–69.1)
Niue	156.6	(132.1–182.7)	55.4	(50.2–76.1)
Northern Mariana Islands	147.6	(122.8–175.6)	58.6	(48.6–65.3)
Palau	151.7	(125.0–183.1)	64.6	(53.1–76.6)
Puerto Rico	164.6	(141.1–187.2)	60.9	(43.3–70.9)
Saint Kitts and Nevis	228.8	(195.5–264.1)	115.8	(102.6–135.7)
San Marino	94.0	(78.0–112.6)	37.3	(31.1–48.5)
Tokelau	148.2	(123.5–174.8)	29.9	(26.5–35.8)
Tuvalu	132.1	(108.6–159.4)	36.4	(32.8–41.2)
United States Virgin Islands	195.7	(160.5–240.5)	102.1	(81.9–118.9)
South Sudan	42.0	(34.4–51.6)	19.1	(15.4–24.2)
Sudan	141.2	(116.2–166.2)	72.8	(60.4–82.9)

Age is strongly correlated with PD prevalence. Analysis of GBD 2023 data revealed a pronounced increase in ASPRs, rising from 0.27 per 100,000 males and 0.18 females at ages 20–24 years to 3006.4 and 2102.8 in those aged 95 years and above [35, 38]. It pronounced age gradient underscores the strong association between aging and PD burden, emphasizing the necessity of developing age‑specific strategies for disease surveillance, early diagnosis, and long‑term care planning.

Significant sex differences are observed in PD prevalence, with ASPR of 152.88 per 100,000 in males compared with 108.93 per 100,000 in females, yielding a male‑to‑female ratio ranging from 1.4 to 1.7 [35, 38] (see Table 1). Males consistently demonstrate higher prevalence across all age groups, with the disparity most pronounced between 50 and 70 years of age [40]. However, recent meta‑analyses suggest that the magnitude of gender disparity may be smaller than traditionally reported [39, 41]. For instance, Zirra et al. reported a global male‑to‑female prevalence ratio of only 1.18 (95% CI: 1.03–1.36) [41], indicating that “gender differences in PD prevalence may not be as pronounced as previously thought,”—displaying a potential narrower gap and highlighting the need to explore methodological, regional, or diagnostic influences on observed differences.

Looking ahead, a modeling study published in *The BMJ* in 2025 projects that the global number of individuals living with PD will reach 25.2 million by 2050 —representing a 112% increase from 2021. The most substantial growth is anticipated among older adult populations and countries with middle‑SDI levels [42]. This projected surge underscores a mounting public health challenge that will extend beyond patients to affect families, caregivers, communities, and health systems worldwide.

### 2.2 Longitudinal Trends Over the Past 50 Years

Over the past five decades, the consensus of PD has undergone significant shifts across epidemiological and clinical domains. During the 1970s and 1980s, systematic data on PD remained scarce, particularly in low‑ and middle‑income regions. Available cohort studies from that era indicate that diagnosis typically occurred before the age of 65 years [43], a pattern likely influenced by both smaller elderly populations and limited availability of sensitive early‑detection methods [44]. Therapeutic options were restricted, and median survival of approximately 9.4 years for patients diagnosed before the age of 70 years, based on data from the pre‑levodopa era [45]. The absence of standardized diagnostic criteria further contributed to substantial underdiagnosis or delayed recognition, especially for nontremor or atypical presentations [46].

During the 1990s to 2000s, the widespread adoption of levodopa‑based therapies and growing disease awareness contributed to improved survival and higher detection rates of PD [45, 47]. However, these benefits remained concentrated in high‑income countries with robust health systems [48]. Concurrently, global age‑standardized incidence of PD also increased [47], a trend driven in part by demographic aging and refinements in diagnostic practice [49]. Despite these improvements, significant diagnostic delays persisted, particularly those patients with predominantly nonmotor or atypical symptoms [50].

From 2010 to the early 2020s, PD demonstrated continued global growth, with the most rapid increases in the incidence observed in middle‑SDI countries, reflecting both population aging and expanded diagnostic reach [38, 51]. Clinical awareness expanded to include nonmotor symptoms, but early diagnosis and longitudinal care remained constrained in resource‑limited settings [52]. In the United States, late‑stage PD was often diagnosed at a mean age of 78 years [53], while in South Korea, only 46% of patients diagnosed in the mid‑2000s survived beyond 10 years [54]. These figures underscore the persistent challenges in achieving timely detection and invention. Meanwhile, the global disability burden intensified, with years lived with disability rising sharply by 2021—a trend largely driven by cognitive impairment, psychiatric symptoms, and loss of independence [51, 53, 55].

Taken together, PD has undergone a distinct epidemiological transition over the past five decades, from early mortality and under‑recognition toward later diagnosis, prolonged survival, and extended disability duration. This evolution represents a transition from acute intervention to the growing complexities of long‑term, multidisciplinary care.

### 2.3 Reflections and Future Directions in PD Epidemiology

Epidemiological data reveal a growing global burden of PD, exhibiting significant heterogeneity across age groups, sexes, and geographic regions. Prevalence rises steeply with age and remains consistently higher in males [35, 38, 40, 42], though recent studies suggest this gap may be narrowing [41]. Geographic disparities are pronounced—North Africa and the Middle East show the highest prevalence, while data remain sparse in low‑income regions [35, 38]. Longitudinal trends show a five‑decade transition: from early underdiagnosis and limited therapeutics toward improved survival and broader symptom recognition, especially in high‑income countries [43, 44, 45, 47, 48]. Yet, diagnostic delays persist, particularly in resource‑limited settings [50, 52, 54]. Together, these findings underscore the urgent need for enhanced global surveillance, earlier detection capabilities, and more equitable care delivery systems [42].

Together, current trends reflect not only increasing PD prevalence but also an extended duration of functional impairment and disability [51, 53, 55]. However, critical gaps persist: many underdeveloped regions lack robust surveillance, nonmotor symptoms remained underreported, and sex‑disaggregated data show inconsistencies [40, 41, 52, 56]. Moving forward, priorities should include expanding epidemiological coverage in low‑resource settings, standardizing diagnostic criteria, and improving long‑term care access [46, 52, 54]. Future efforts should address not only disease burden, but also quality of live through better data collection, more timely diagnosis, and more equitable delivery of person‑centered care [42, 52].

## 3 Risk Factors

**FIGURE 1:**
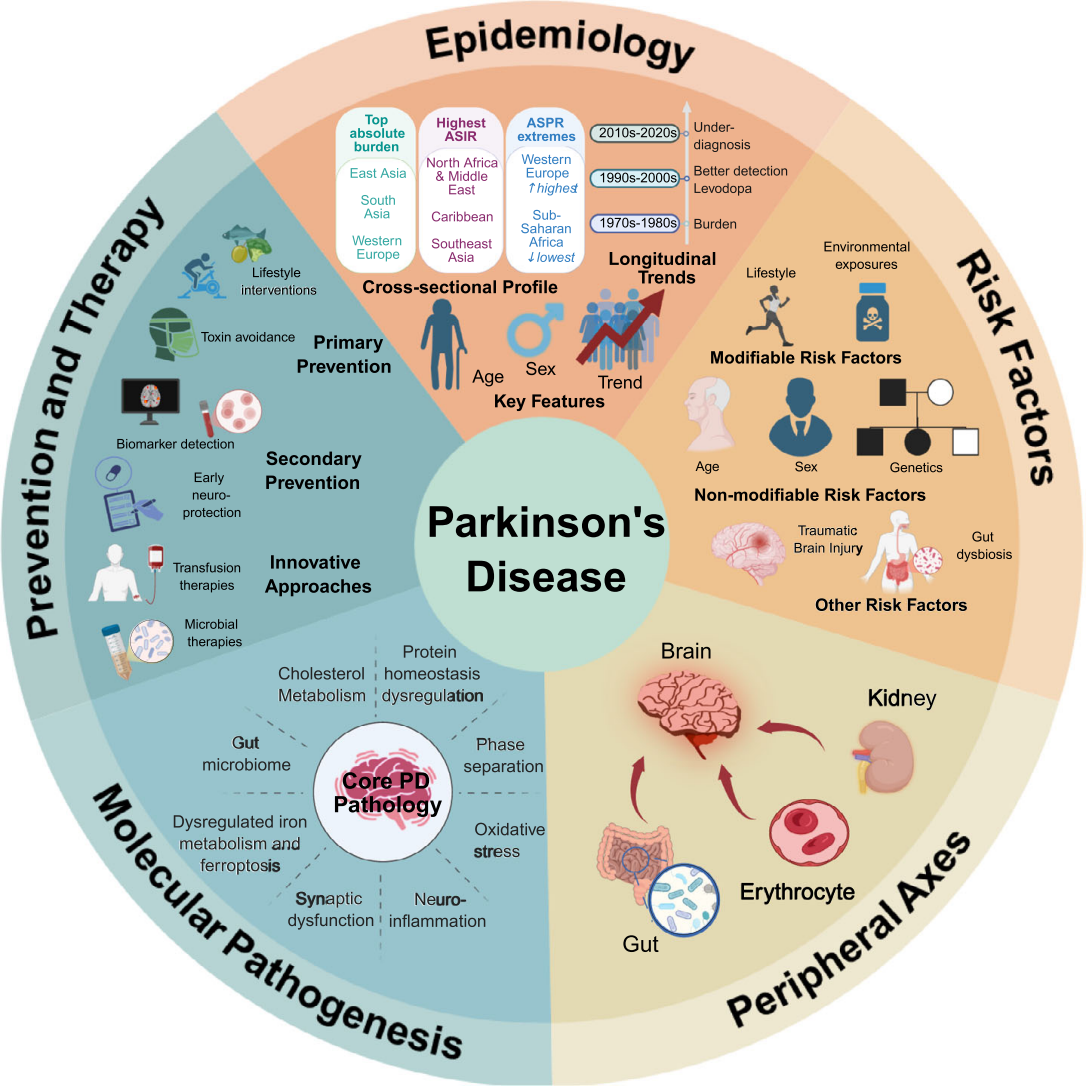
Parkinson's disease: a multidimensional overview of epidemiology, risk factors, pathogenesis, and therapeutic strategies. This graphical abstract summarizes the major components of this review on Parkinson's disease, including epidemiological trends, risk factor classification, peripheral and molecular pathogenesis, and both preventive and therapeutic strategies. The framework reflects the multidimensional organization of the text, covering disease burden, mechanistic insights, and intervention perspectives. Created in BioRender. Guo, C. (2025) https://BioRender.com/y6yb9vl.

**FIGURE 2:**
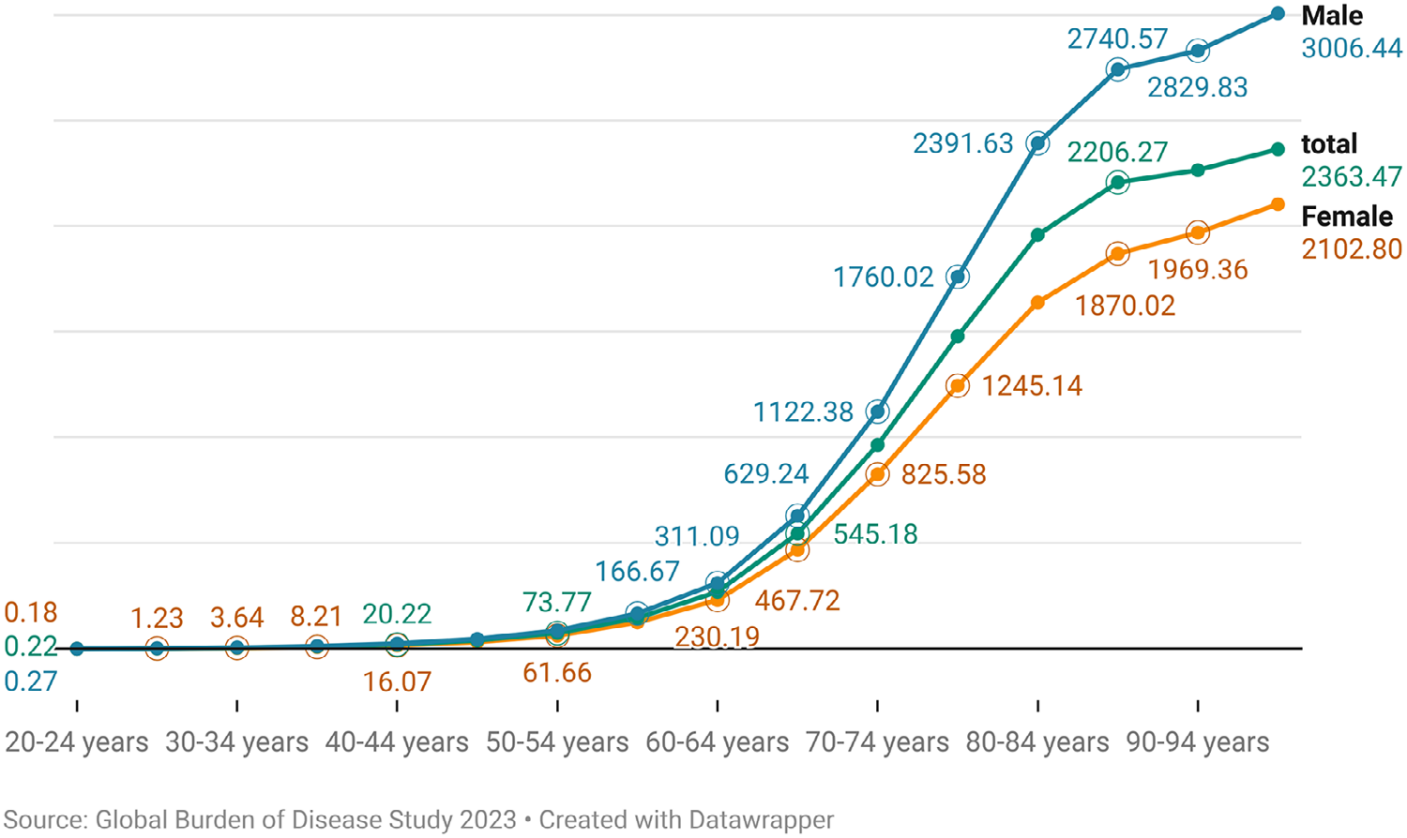
Global age‐specific PD prevalence curves (male, female, total). Age‐specific prevalence of Parkinson's disease is plotted across seven age groups from 20–24 to ≥95 years, based on Global Burden of Disease Study 2023 data. Three curves represent total, male, and female prevalence (per 100,000 population). Prevalence is lowest in the 20–24 years group and increases with age, peaking at ≥95 years. (total = 0.22 → 2363.47; female = 0.18 → 2102.80; male = 0.27 → 3006.44). Males consistently show higher prevalence than females across all age groups.

**FIGURE 3:**
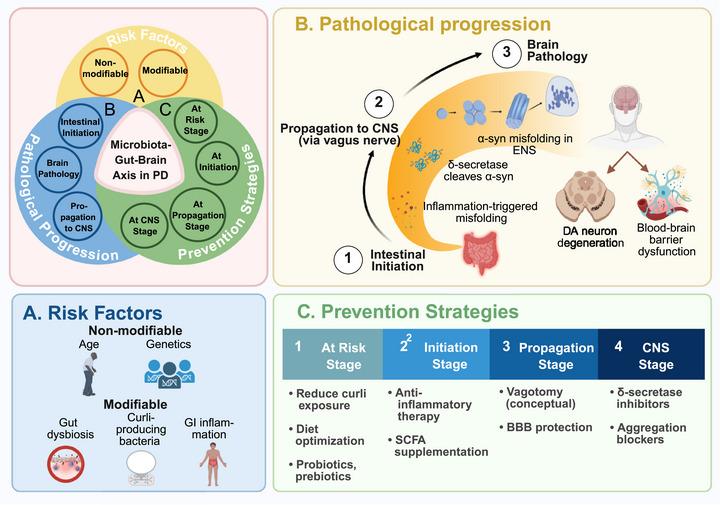
The microbiota–gut–brain axis in Parkinson's disease: risk factors, pathological progression, and prevention strategies. This figure summarizes three major aspects of Parkinson's disease (PD) within the framework of the microbiota–gut–brain axis. The diagram in the upper left illustrates their conceptual relationship, indicating that risk factors, pathological progression, and prevention strategies all originate from this shared biological pathway. (A) Risk factors associated with the microbiota–gut–brain axis, categorized as nonmodifiable (e.g., age, sex, genetic susceptibility) and modifiable (e.g., gut dysbiosis, bacterial amyloids, gastrointestinal inflammation, environmental exposures), which interact with the axis to influence PD susceptibility. (B) Key stages of pathological progression, initiating in the gut with α‐synuclein misfolding and local immune activation, followed by retrograde propagation to the central nervous system, and ultimately resulting in brain pathology (e.g., dopaminergic neurodegeneration in the substantia nigra, blood–brain barrier dysfunction). (C) Prevention strategies tailored to specific disease stages, including early interventions during the at‐risk phase, microbiota‐targeted and anti‐inflammatory approaches during initiation and propagation, and neuroprotective strategies during CNS involvement. Created in BioRender. Guo, C. (2025) https://BioRender.com/3au2rc6.

Identifying risk factors for PD is crucial for elucidating its pathogenesis and informing prevention strategies. This section focuses on three categories of risk factors: nonmodifiable factors (such as age, sex, and genetics), modifiable factors (including environmental exposures and lifestyle variables), and emerging contributors like traumatic brain injury (TBI) and gut microbiome dysbiosis. By systematically evaluating these factors, we aim to clarify their contributions to PD susceptibility and highlight promising avenues for targeted intervention (Figures 1, 2, 3).

### 3.1 Non‐modifiable Risk Factors

Age constitutes the most well‑established nonmodifiable risk factor for PD, with multifaceted cellular aging processes fundamentally contributing to dopaminergic neuron vulnerability. Advancing age progressively impairs critical intracellular systems [57]. First, mitochondrial dysfunction manifests as the accumulation of mtDNA mutations [57, 58], disrupted energy metabolism [59], and elevated reactive oxygen species (ROS) production [60], severely affecting the energy‑dependent substantia nigra dopaminergic neurons [61]. Second, proteostasis disruption involves declined autophagy–lysosomal and ubiquitin–proteasome activity, rendering proteins like α‑syn prone to aggregation and resistant to clearance [62]. Furthermore, a chronic inflammatory state in the nervous system intensifies with age, leading to increased microglial activation and elevated levels of proinflammatory cytokines, such as tumor necrosis factor‑alpha (TNF‑α) and interleukin‑1 beta (IL‑1β), detectable even preclinically [63, 64].

Compared with chronological age, recent evidence suggests that biological age, assessed via epigenetic markers, may more accurately reflect the molecular trajectory of PD [65]. Epigenetic clocks based on DNA methylation patterns have revealed accelerated epigenetic ageing in PD patients, suggesting a potential role for age‑related transcriptional dysregulation in disease pathogenesis [65, 66].

Sex differences are evident in both the pathogenesis and clinical manifestations of PD. In females, high estrogen levels provide neuroprotection by reducing oxidative stress, inflammation, and dopaminergic neuron loss [67, 68, 69, 70]. Furthermore, females exhibit greater baseline availability of dopamine transporter (DAT) proteins, potentially enhancing neuronal resilience against neurotoxic challenges [71, 72]. In contrast, early androgen exposure intricately shapes male brain circuits in ways that significantly increase vulnerability to neurodegeneration [73, 74]. Studies also show that male brains mount stronger glial inflammatory responses under stress, exacerbating dopaminergic damage [75]. These sex‑based hormonal and developmental distinctions likely contribute to the observed disparity in PD risk, suggesting that biological sex should be considered in PD risk stratification and mechanistic research [76].

Genetic factors contribute to PD through both monogenic and polygenic mechanisms [77, 78]. Monogenic mutations in genes such as *SNCA*, *LRRK2*, and *PARK2* provide key PD pathological mechanisms, including α‑syn aggregation and impaired mitophagy [79, 80]. In contrast, polygenic risk stems from the cumulative effect of common variants identified in genome‑wide association studies (e.g., *GBA*, *MAPT*, *TMEM175*), which often interact with environmental triggers [81, 82, 83]. Collectively, genetic research enhances our understanding of PD heterogeneity and supports the development of precision medicine approaches [84].

### 3.2 Modifiable Risk Factors

Environmental exposures constitute significant modifiable risk factors for PD. Epidemiological evidence shows that chronic exposure to the herbicide paraquat (PQ) elevates PD risk by approximately 64% [85]. This robust association is frequently attributed to PQ‑induced oxidative stress, a mechanism extensively documented in both in vitro and in vivo models [86, 87]. Functioning as a redox cycling agent, PQ generates ROS within dopaminergic neurons, leading to mitochondrial dysfunction and lesions in the SNpc [87, 88]. Rotenone, another widely studied pesticide, is known to induce PD‑like symptoms. Its high lipophilicity allows easy crossing of biological membranes, including the BBB, resulting in accumulation in the CNS [89]. Intracellularly, rotenone primarily inhibits mitochondrial complex I, disrupting oxidative phosphorylation and impairing proteasomal function [90]. Beyond pesticides, heavy metals such as iron and manganese are also well‑recognized risk factors for PD. Iron catalyzes oxidative damage via the Fenton reaction, while manganese accumulates in dopaminergic neurons and exerts neurotoxic effects [91, 92]. Air pollution, particularly exposure to fine particulate matter, has also been implicated. Cohort studies published in *Environmental Health Perspectives* report significantly elevated PD incidence among populations with high PM2.5 exposure [93]. Experimental models further demonstrate that PM2.5 disrupts BBB integrity and activates microglia [94, 95]. Emerging data from animal and cellular studies suggest that microplastics may contribute to neuroinflammation and oxidative stress, though epidemiological data in humans remain limited [96, 97].

Lifestyle factors, particularly diet and physical activity, are increasingly recognized as modifiable elements that may influence PD risk. Dietary patterns are increasingly recognized as modifiable risk factors for PD, acting through mechanisms such as oxidative stress, chronic inflammation, and gut microbiota dysregulation [98]. Diets high in saturated fats and heme iron, which commonly derived from animal products such as red meat, are associated with elevated PD risk, likely by inducing mitochondrial dysfunction and promoting oxidative damage [99]. Excessive dairy consumption has also shown a consistent positive association with PD incidence in multiple cohort studies [100, 101]. It is hypothesized that dairy intake may reduce levels of uric acid, an endogenous antioxidant, or introduce neurotoxic contaminants [102, 103]. In contrast, the Mediterranean diet, characterized by abundant fruits, vegetables, legumes, whole grains, and olive oil, is associated with reduced PD risk [104, 105]. Its neuroprotective potentials include enhanced antioxidant defenses, improved metabolic homeostasis, and beneficial gut microbiome modulation [106, 107, 108]. While causality remains to be established, these findings highlight the important role of diet in PD susceptibility.

Physical inactivity and sedentary behavior are established risk factors for PD. While regular physical activity confers neuroprotection through multiple pathways [109], emerging evidence indicates that sedentary behavior independently contributes to PD risk, possibly via chronic systemic inflammation and reduced cerebral perfusion [110, 111, 112, 113]. Further evidence shows that physical activity promotes neural resilience by upregulating brain‑derived neurotrophic factor (BDNF) and supporting corticostriatal circuitry [114, 115, 116]. These findings collectively support the role of physical activity not only as a promoter of general health, but also as a modifiable factor for reducing PD incidence.

### 3.3 Other Risk Factors

In addition to the above‑mentioned risk factors, TBI and gut dysbiosis represent additional risk factor for PD, potentially through overlapping mechanisms involving chronic neuroinflammation and the aggregation of misfolded proteins. Epidemiological studies consistently demonstrate an elevated risk of PD following TBI. A cohort study found that individuals aged 55 years and older with TBI had a 44% higher risk of developing PD compared with those who experienced noncranial trauma [117]. Similarly, a 2023 meta‑analysis reported a pooled relative risk of 1.48 for PD following TBI across 15 studies [118]. Proposed mechanisms underlying this association include microglial activation, disruption of dopaminergic pathways, and accelerated α‑syn aggregation [119].

Parallel evidence highlights the role of gut dysbiosis in PD pathogenesis. Longitudinal studies show that gastrointestinal symptoms such as constipation may precede motor manifestations by up to two decades, supporting the involvement of gut–brain axis [120]. Meta‑analyses have identified specific microbial dysbiosis in PD patients, including decreased abundance of short‑chain fatty acid (SCFA)‑producing genera (e.g., *Prevotella*) and increased abundance of proinflammatory *Enterobacteriaceae* [121]. Experimental evidence further demonstrates that fecal microbiota transplantation (FMT) from PD patients into mice exacerbated motor deficits and neuroinflammation [122].

The pathogenesis of PD arises from a complex interplay of nonmodifiable and modifiable risk factors, as well as emerging contributors such as TBI and gut microbiota dysbiosis. These risk factors not only influence individual susceptibility but may also shape the trajectory and pace of disease progression. A better understanding of how such diverse influences trigger and interact with downstream pathological changes is essential for uncovering the full spectrum of PD pathogenesis. Moreover, recognizing the modifiable nature of certain risk factors has significant implications for early prevention and intervention. The following sections will explore the peripheral and molecular mechanisms underlying PD, while also establishing the foundation for mechanism‑based preventive and therapeutic strategies.

## 4 Pathogenesis in PD

PD pathogenesis is increasingly understood as a complex, multisystem process involving interactions beyond the CNS. Emerging evidence highlights several peripheral‑to‑central axes that contribute to disease initiation and progression. Among these, the gut–brain axis implicates gastrointestinal dysfunction and microbial dysbiosis in triggering pathological α‑syn aggregation that may propagate to the brain. Concurrently, the erythrocyte–brain axis suggests that peripheral blood components, particularly erythrocyte‐derived α‑syn and extracellular vesicles (EVs), influence central neurodegeneration. Furthermore, the kidney–brain axis reflects the role of renal impairment in exacerbating neuroinflammation, metabolic imbalance, and toxin accumulation relevant to PD pathology. The following sections will delve into the peripheral and molecular mechanisms underlying PD, thereby establishing a foundation for mechanism‑driven preventive and therapeutic approaches.

### 4.1 Gut–Brain Axis

A growing body of evidence suggests that PD may originate in the gut, with pathological α‑syn aggregation potentially initiating in the enteric nervous system before spreading to the brain. Braak et al. first demonstrated α‑syn‑positive inclusions within the Meissner's and Auerbach's plexuses of the gastrointestinal tract prior to their detection in the CNS, proposing caudo‐rostral propagation via the vagus nerve [19]. Supporting this, Lebouvier et al. identified similar α‑syn accumulations in colonic biopsies from prodromal PD patients, further strengthening the hypothesis of a gut‐initiated pathological process [20]. Recent advances in molecular imaging further support this propagation pattern. Using the α‑syn‑specific PET tracer [^18^F]‑F0502B, Xiang et al. successfully mapped the sequential spread of α‑syn from the gut to the brain in a mouse model, providing direct evidence for gut‑to‑brain α‑syn transmission [21].

Functional studies further demonstrate that gut microbiota may contribute to PD‑relevant phenotypes. For example, Sampson et al. transplanted fecal microbiota from PD patients into germ‑free mice resulted in worsened motor function and enhanced neuroinflammation. Conversely, antibiotic‑mediated microbiota depletion alleviated these impairments, suggesting a causal link between gut microbial composition and PD neuropathology [22]. Complementary human microbiome analyses by Scheperjans et al. identified a distinct dysbiotic profile in PD patients, characterized by decreased *Prevotellaceae* and increased *Enterobacteriaceae—*with the latter correlating positively with motor symptom severity [123]. These microbial shifts have been corroborated in independent cohorts, and multiomics analyses further indicate alterations in microbial gene expression and metabolite production in PD patients [124].

Building on the evidence linking gut microbiota to PD, recent studies have explored how dysbiosis may contribute to the initiation and propagation of α‑syn pathology. For instance, a microbiome‑wide association study by Payami et al. identified an increased abundance of opportunistic pathogens in the gut microbiome of PD patients [25], which are proposed to incite intestinal inflammation and facilitate α‑syn misfolding [125]. Misfolded α‑syn forms oligomers and fibrils that ultimately develop into LBs [9]. A notable example is *Escherichia coli* (*E. coli*), which produces the bacterial amyloid curli that facilitates α‑syn aggregation in both the gut and brain, accelerating neuronal damage [10]. Additionally, gut dysbiosis also disrupts microbial metabolism and immune balance. It reduces beneficial metabolites, such as SCFAs, while increasing proinflammatory cytokine production. These changes collectively contribute to a chronic inflammatory state. SCFAs typically exert anti‑inflammatory effects by activating bile acid receptors and regulating oxidative stress; however, PD patients often exhibit reduced SCFA levels [126, 127]. Concurrently, microbial‑derived cytokines can activate local intestinal immune responses and promote neuroinflammation via the gut–brain axis [128, 129]. Clinical cohort studies further strengthen this connection, revealing a higher incidence of PD among individuals with Crohn's disease compared with matched controls [130, 131]. Furthermore, studies using DSS‑induced colitis models demonstrated significantly increased α‑syn expression in the colon and notable α‑syn deposits in the brain [132], supporting the hypothesis that the gastrointestinal tract serves as an initial site for α‑syn pathology, which may then propagate to the CNS via the vagus nerve [23]. Notably, vagotomy experiments demonstrate that interrupting this pathway effectively blocks the spread of PD pathology from gut to brain [133].

More recently, δ‑secretase (asparagine endopeptidase) has been identified as a key enzymatic contributor to this process [26]. It cleaves both α‑syn and Tau protein at specific sites, generating truncated fragments that exhibit heightened propensity for toxic fibril formation [27]. In rotenone‑induced PD models, chronic gut inflammation was shown to activate δ‑secretase, accelerating the formation of α‑syn and Tau into fibrils capable of migrating along the vagus nerve and contributing to the loss of dopaminergic neurons. Notably, this pathogenic cascade was markedly in δ‑secretase‑deficient animals [26, 27]. Experimental studies further show that these fibrils are structurally stable and highly neurotoxic, their introduction into the colon reproduced key motor and cognitive deficits associated with PD [134, 135], underscoring the role of δ‑secretase as a molecular link between peripheral inflammation and central neuropathology.

In summary, gut dysbiosis may contribute to PD pathogenesis through multiple mechanisms, including pathogen‑driven inflammation, metabolic alterations, immune activation, and enzyme‑mediated proteotoxicity. These pathways support the concept of a gut‑to‑brain propagation model in PD.

### 4.2 Erythrocyte–Brain Axis

Emerging evidence increasingly supports a contributory role for erythrocytes in PD pathogenesis. Notably, erythrocytes represent the predominant peripheral reservoir of α‑syn, harboring levels up to 1000‑fold higher than those found in cerebrospinal fluid (CSF) [28]. Studies report significantly elevated concentrations of both total and oligomeric α‑syn in the erythrocytes of PD patients compared with healthy controls [29]. Given that erythrocytes are the major peripheral source of α‑syn, such changes have attracted attention in studies of early PD‑related alterations [136]. Furthermore, several studies have observed abnormalities in erythrocyte antioxidant enzyme activity in PD. Compared with matched controls, PD patients exhibit reduced levels of superoxide dismutase (SOD), catalase, and glutathione (GSH) peroxidase (GPX). These alterations often correlate with clinical severity [137]. While these findings do not clarify whether oxidative stress is a cause or consequence of PD, they reinforce the presence of systemic biochemical alterations involving erythrocytes. In MPTP‑induced mouse models of PD, structural and functional changes in erythrocyte membranes, including altered lipid microviscosity and disrupted enzyme activity, have been detected during early disease stages [138]. Importantly, erythrocyte‑derived EVs from PD patients have been shown to cross the BBB in vivo and localize to brain regions relevant to PD pathology [30].

Building on observations of α‑syn species and EVs originating from erythrocytes in PD patients, emerging evidence suggests a potential erythrocyte‑to‑brain transmission route. This section outlines proposed mechanisms by which erythrocyte‑originated molecular components may interact with the CNS and influence disease progression. A prominent hypothesis involves the release of erythrocyte‑derived EVs containing α‑syn, particularly in its oligomeric form. These vesicles can cross the BBB and accumulate within microglia and astrocytes, potentially disrupting glial homeostasis. Animal model studies indicate that such vesicles localize to astrocytic endfeet and impair glutamate uptake by modulating EAAT2 function, indicating a possible contribution to excitotoxic stress [28]. Proteomic profiling further reveals that the cargo composition of erythrocyte‑derived EVs correlates with disease severity, suggesting a potential link between vesicle content and functional impact in the brain [31]. Beyond vesicle content, the biogenesis and release of erythrocytes appears significantly influenced by oxidative stress, which is frequently reported in the peripheral blood of PD patients. Diminished activity of antioxidant enzymes (e.g., SOD, catalase) in PD erythrocytes may facilitate increased vesicle shedding and influence the redox state of vesicle cargo [139]. Moreover, oxidative modification to vesicle membranes enhances their uptake by glial cells and increases their biological activity [140]. Lipid peroxidation products within these vesicles also contribute to impaired mitochondrial function in neuronal systems, highlighting a potential mechanism of toxicity following vesicle entry into the brain [141].

A further dimension involves the role of erythrocytes in systemic iron metabolism, frequently dysregulated in PD. Alterations in erythrocyte iron transport proteins may increase iron loading within vesicles, modifying their oxidative profile. Experimental evidence indicates that iron‑rich EVs promote α‑syn aggregation and enhance inflammatory responses by glial cells [142]. Additionally, iron‐dependent redox reactions within these vesicles have been proposed to enhance BBB penetration via oxidative stress‑mediated processes [143]. Correlative studies show that iron‑related markers in circulating EVs track with PD severity, although further investigation is required to establish their functional role in disease initiation or progression [144].

Collectively, these findings support the hypothesis that erythrocyte‑derived vesicles act as a conduit for peripheral pathological signals to reach and influence the CNS. While this axis remains under investigation, current data provide a conceptual framework for future research on erythrocyte‑mediated molecular communication in neurodegenerative diseases.

### 4.3 Kidney–Brain Axis

As PD is increasingly recognized as a systemic disorder extending beyond the CNS, accumulating evidence highlights the potential involvement of renal dysfunction in PD pathogenesis and progression. This has given rise to the concept of a kidney–brain axis, describing how impaired kidney function may influence neurodegenerative processes in the brain [32]. A large‑scale nationwide cohort study from South Korea demonstrated that reduced estimated glomerular filtration rate (eGFR) and proteinuria were significantly associated with increased PD incidence, even after adjustment for comorbidities such as hypertension and diabetes [145]. Similarly, a retrospective analysis of health data from Taiwan, China revealed a 73% higher PD risk in patients with end‑stage renal disease (ESRD) compared with the general population [146]. Beyond disease onset, impaired kidney function may also reflect clinical progression in PD patients. Qu et al. reported that longitudinal declining eGFR correlated with cognitive deterioration and abnormal neurodegenerative biomarkers in PD patients, indicating its potential as a prognostic indicator [147]. Complementing these findings, a case–control study showed significantly elevated serum creatinine, blood urea nitrogen (BUN), and urinary protein levels in PD patients, which positively correlated with Hoehn‑Yahr stage and motor symptom severity [148].

In addition to these epidemiological and clinical observations, experimental confirmation in preclinical PD models, demonstrated that renal dysfunction directly promotes neurodegeneration by impairing the clearance of circulating α‑syn, thereby validating the kidney–brain axis hypothesis [149].

Beyond conventional renal function indices, specific protein‑based markers have also been implicated in PD. Serum and urinary β_2_‑microglobulin levels are elevated in PD patients, particularly those with longer disease duration, suggesting its potential value as a progression‑related biomarker [150]. Proteinuria has further been hypothesized to reflect systemic endothelial dysfunction, oxidative stress, and insulin resistance, which may indirectly contribute to PD pathophysiology [151]. Collectively, these findings highlight a spectrum of renal biomarkers, including eGFR, creatinine, BUN, β_2_‑microglobulin, and albuminuria, that may provide clinically meaningful insights into PD risk and progression, and pave the way for mechanistic exploration of the kidney–brain axis in PD.

Pathological α‑syn deposits have been identified in the kidneys of patients with LB disease and ESRD. The healthy kidney clears circulating α‑syn while renal failure reduces this clearance and leads to renal deposition and subsequent spread of α‑syn to the brain. In rodent models, intrarenal injection of α‑syn preformed fibrils induced renal deposits and triggered propagation of pathology to the CNS. Notably, renal denervation prevented this propagation, spread, and reductions in peripheral α‑syn were shown to ameliorate central pathology, thereby demonstrating the dependence of CNS involvement on peripheral α‑syn. While these findings strongly support a peripheral–origin, trans‑organ propagation pathway connecting renal dysfunction to PD, it is important to note that they are derived from experimental seeding models. Consequently, direct extrapolation to the majority of sporadic human PD cases should be made cautiously [149].

Mounting evidence suggests that renal dysfunction may influence PD neurodegeneration through several interrelated biological pathways. One proposed mechanism involves impaired clearance of protein‑bound uremic solutes, which are normally excreted by the kidneys. Metabolites such as indoxyl sulfate and p‑cresol sulfate, when BBB integrity is compromised, can interacted with glial cells to promote oxidative stress, proinflammatory cascades, and mitochondrial dysfunction within the CNS. These changes can lower the threshold for pathogenic α‑syn misfolding and aggregation, thereby exacerbating neurodegeneration [33]. Notably, several uremic solutes have also been implicated in systemic endothelial dysfunction and microvascular injury, suggesting a link to the proteinuria‑related cerebrovascular changes observed in epidemiological PD studies [145]. Beyond impaired toxin clearance, declining kidney function is accompanied by chronic systemic inflammation, with elevated circulating levels of cytokines (e.g., IL‑6, TNF‑α, CRP) [34]. These inflammatory mediators have been shown to modulate BBB permeability and activate microglia, leading to a sustained neuroinflammatory environment that increases neuronal vulnerability, and potentially facilitating peripheral α‑syn aggregation and spread [152, 153]. Furthermore, inflammation‑driven endothelial injury and autonomic dysregulation may integrate renal pathology into broader pathophysiological networks, such as the brain–heart–kidney axis [154]. Metabolic dysregulation represents a third pathway linking renal impairment to PD progression. Disturbances in calcium–phosphate metabolism, acid–base balance, and vitamin D synthesis are common in chronic kidney disease and have been linked to altered synaptic function, excitotoxicity, and reduced dopaminergic neuronal resilience [33]. For instance, vitamin D deficiency enhances oxidative stress and impairs mitochondrial dynamics, both implicated in PD pathology [155]. These metabolic alterations may also intersect with systemic insulin resistance and oxidative burden, reinforcing the hypothesis that renal dysfunction acts as a metabolic amplifier in PD progression.

Collectively, the gut–brain, erythrocyte–brain, and kidney–brain axes highlight the systemic complexity of PD pathogenesis, where peripheral dysfunction contributes to central neurodegeneration through diverse molecular and cellular pathways. These insights emphasize that PD is not solely a brain disorder but involves multisystem interactions that influence disease onset and progression. Recognizing these axis‑related mechanisms deepens understanding of PD etiology and identifies potential peripheral biomarkers and novel therapeutic targets linked to these systemic pathways. Such mechanistic insights provide a foundation for further exploration of prevention and therapeutic strategies aimed at mitigating risk factors and intervening in these pathogenic processes.

## 5 Molecular Pathogenesis

Building upon the systemic perspective of the previous section, this section examines key cellular pathways involved in PD molecular pathogenesis. These interrelated pathological processes include protein homeostasis dysregulation, mitochondrial dysfunction, oxidative stress, neuroinflammation, synaptic impairment, and metabolic disturbances such as iron and cholesterol dysregulation. Together, they drive α‑syn misfolding, aggregation, and neuronal degeneration. By elucidating these interconnected pathways, this section complements the broader systemic view and highlights potential targets for therapeutic intervention.

### 5.1 Protein Homeostasis Dysregulation

Under pathological conditions, α‑syn, a natively unfolded presynaptic protein, misfolds and aberrantly aggregates into toxic oligomeric and fibrillar species. These misfolded aggregates evade normal protein quality control mechanisms and progressively accumulate within neurons. Among these, α‑syn oligomers (α‑synOs) are considered highly neurotoxic due to their ability to induce mitochondrial dysfunction, impair synaptic transmission, damage cellular membranes, and trigger intracellular inflammation and lysosomal stress [11]. Moreover, their interaction with membrane‑associated receptors, such as prion proteins and β‑spectrin, further exacerbates oxidative stress, elevates ROS levels, reduces ATP production, and disrupts cytoskeletal dynamics [156, 157, 158].

Importantly, misfolded α‑syn species exhibit prion‑like intercellular propagation [159]. Once released from affected neurons, α‑synOs can be internalized by neighboring cells, including neurons, astrocytes, and microglia—triggering the misfolding of endogenous α‑syn and perpetuating pathological aggregation [160]. Microglia recognize α‑synOs through pattern recognition receptors such as Toll‑like receptor (TLR)2, triggering activation of the NLRP3 inflammasome and a proinflammatory cascade [161]. Under chronic conditions, the neuroinflammatory response fosters a toxic microenvironment that exacerbates α‑syn pathology. Furthermore, persistent inflammasome activation also disrupts autophagic regulation in glial cells, impairing the clearance of aggregated proteins and contributing to the loss of neuroimmune homeostasis [162, 163, 164]. Transgenic models provide further support for these mechanisms. For instance, the M83 mouse expressing mutant human α‑syn demonstrates that abnormal α‑syn accumulation is sufficient to drive neurodegenerative phenotypes including neuronal hyperactivity and vascular patterning defects, even in the absence of overt systemic inflammation [164, 165]. Collectively, these findings highlight the intrinsic toxicity and self‑perpetuating propagation of misfolded α‑syn as a core mechanism disrupting proteostasis in PD.

While the aggregation and propagation of misfolded α‑syn contribute to neurotoxicity in PD, its persistence within affected neurons also points to a fundamental impairment of intracellular protein clearance mechanisms. This section explores the compromised function of two crucial degradative pathways essential for protein homeostasis: the ubiquitin–proteasome system (UPS) and the autophagy–lysosome pathway (ALP).

In PD, disruption of proteostasis is strongly linked to dysfunction in both the UPS and ALP. The UPS eliminates short‑lived and misfolded proteins via ubiquitination followed by degradation by the 26S proteasome. In PD, aggregated α‑syn, particularly in its protofibrillar form, impairs proteasome activity, reducing its efficiency in processing both ubiquitinated and nonubiquitinated substrates [166, 167, 168]. Genetic factors further compound this dysfunction. Mutations in UPS‑related genes, such as *parkin* and *UCH‑L1*, compromise ubiquitin tagging or removal, hindering substrate recognition and clearance [169]. Additionally, environmental stressors like heavy metals and pesticides have been shown in experimental models to impair proteasome function, promote α‑syn aggregation, and drive dopaminergic neuron loss, underscoring the multifaceted contribution of UPS impairment to PD pathogenesis [170].

Parallel to UPS dysfunction, the ALP is also significantly affected in PD. The ALP is also substantially compromised in PD. The ALP mediates the clearance of larger protein aggregates and damaged organelles through macroautophagy and chaperone‑mediated autophagy (CMA). Under physiological conditions, wild‑type α‑syn is efficiently degraded via CMA. However, mutant variants such as A53T bind to the lysosomal receptor LAMP‑2A but fail to translocate into the lumen, resulting in a blockage that impairs not only their own degradation but also that of other CMA substrates [171, 172]. Moreover, mutations in *GBA* impair lysosomal enzyme activity and acidification, leading to defective autophagic flux [173]. α‑syn aggregates are also implicated in blocking autophagosome–lysosome fusion, a critical step in macroautophagy. Additionally, exposure of microglia to extracellular α‑synOs triggers inflammasome activation and disrupts autophagic regulation, potentially impairing immune surveillance and exacerbating neuroinflammation [174].

Importantly, UPS and ALP dysfunction are not independent but mechanistically interconnected. When proteasomal capacity is compromised, excess misfolded or aggregated proteins accumulate and are redirected to autophagy for clearance. Conversely, when autophagy is impaired, large aggregates that cannot be processed by the proteasome persist and may further burden the UPS. This reciprocal overload establishes a pathological feedback loop, wherein the failure of one system amplifies the failure of the other. Such crosstalk contributes to chronic proteostasis collapse, persistent α‑syn accumulation, and progressive dopaminergic neuron loss [175, 176].

### 5.2 Phase Separation

Increasing evidence suggests that α‑syn undergoes liquid–liquid phase separation (LLPS) in the early stages of PD, forming condensates that act as nucleation sites for pathological aggregation [177]. LLPS describes the demixing of biomolecules into concentrated, dynamic, membrane‑less liquid droplets. This process is typically mediated by proteins containing intrinsically disordered regions and low‑complexity domains, features prominently exhibited by α‑syn [178]. Under physiological conditions, α‑syn can form such droplets in response to crowding and moderate salt concentrations. These condensates markedly increase local protein concentrations, facilitating multivalent interactions and β‑sheet transitions that drive fibril formation [179].

Although LLPS can occur in healthy neurons, disease‑related stressors increase both its frequency and persistence. Oxidative stress, mitochondrial dysfunction, and impaired protein quality control expose aggregation‑prone motifs and compromise systems maintaining condensate reversibility [180, 181]. For instance, mitochondrial dysfunction, a hallmark of PD, leads to ATP depletion, reducing droplet solubility and accelerating the liquid‑to‑solid transition [182]. These changes are further intensified by acidic pH, divalent metal ions, and familial mutations (e.g., A53T and E46K), which disrupt droplet fluidity and enhance aggregation kinetics [177, 183]. The resulting fibrillar structures form the core of LBs and contribute to neuronal toxicity and degeneration [184].

Dysfunctions in the regulatory systems that govern biomolecular condensate dynamics further exacerbate the pathological consequences of LLPS. ATP functions as a biological hydrotrope, preserving droplet liquidity; its reduction promotes hardening of condensates [182]. RNA and RNA‑binding proteins also play a modulatory role by buffering protein–protein interactions. While most studies suggest RNA exerts a protective effect, some report that certain RNAs may promote condensate aging under stress [185]. In addition, impaired autophagy and chaperone‑mediated disaggregation allow aberrant droplets to persist and evolve [181]. Together, these regulatory deficits reinforce the trajectory toward pathological α‑syn aggregation.

Although LLPS represents a key mechanism for α‑syn condensation and aggregation, it is not the only route involved. Alternative pathways, including direct nucleation on membranes or lipid interfaces, may also contribute to α‑syn misfolding, underscoring the mechanistic complexity underlying PD pathology [179, 185].

### 5.3 Oxidative Stress

One of the principal contributors to oxidative stress in PD is mitochondrial dysfunction, particularly through ROS‑mediated damage. Under physiological conditions, mitochondria regulate intracellular redox homeostasis while producing limited ROS as a byproduct of oxidative phosphorylation. In PD, however, this equilibrium is disrupted. Complex I (NADH: ubiquinone oxidoreductase), the initial enzyme of the mitochondrial electron transport chain, is highly vulnerable to oxidative damage. Postmortem studies consistently revealed Complex I deficiency in the substantia nigra of PD patients. This deficiency impairs ATP synthesis, increases electron leakage, and thereby amplifies ROS generation [60, 186]. As a result, a self‑perpetuating cycle wherein mitochondrial dysfunction enhances ROS production, and elevated ROS further compromises mitochondrial integrity [58]. In experimental models, exposure to mitochondrial toxins such as rotenone or MPP⁺ induces selective dopaminergic neuronal loss through Complex I inhibition, recapitulating key pathological features of PD [187, 188]. Moreover, disruption of mitochondrial quality control pathways, including impaired mitophagy due to PINK1 or Parkin dysfunction, exacerbates oxidative injury and contributes to neuronal vulnerability [58, 189].

In addition to mitochondria‑derived ROS, other intracellular sources also contribute to the oxidative milieu in PD. Dopamine itself can undergo enzymatic and nonenzymatic oxidation, generating hydrogen peroxide and reactive quinones that damage cellular components and promote α‑syn aggregation [190]. Furthermore, chronic activation of microglia releases superoxide through NADPH oxidase and triggers TLR4‑dependent inflammatory cascades, which reinforce ROS accumulation and neurotoxicity [58].

### 5.4 Neuroinflammation

Neuroinflammation in PD develops within the anatomically and immunologically distinct environment of the CNS. This compartment is defined by the presence of the BBB, limited lymphatic drainage, and the reliance on resident immune cells such as microglia. These features constrain peripheral immune access and lead to sustained local immune activation following disruptions to CNS homeostasis. In PD, misfolded α‑syn and neuronal damage‑associated molecules (e.g., ATP, HMGB1) activate microglia through pattern recognition receptors including TLR2 and TLR4 [14], triggering a proinflammatory phenotype with cytokines (TNF‑α and IL‑1β) and ROS release [15].

The neurotoxic effects of activated microglia have been demonstrated in vivo. Intranigral lipopolysaccharide (LPS) injection in rodents induces sustained microglial activation, elevated local TNF‑α and IL‑1β levels, and reduces substantia nigra tyrosine hydroxylase‑positive dopaminergic neurons [13]. These cytokines bind to corresponding receptors on neurons, activating downstream signaling pathways such as NF‑κB and MAPK [24]. Within dopaminergic neurons of the SNpc, these pathways promote oxidative stress, impair mitochondrial dysfunction, and promote apoptotic signaling [13]. Because SNpc neurons are metabolically active and have low intrinsic antioxidant capacity, they are particularly vulnerable to such inflammatory insults [12].

The pathophysiological cascade establishes a self‑perpetuating toxic milieu characterized by neuroinflammation. In experimental models, inhibition of TNF‑α signaling or microglial depletion attenuates dopaminergic neuron loss and preserves motor function [16, 17], demonstrating that neuroinflammation is not a secondary bystander but an active contributor to PD progression.

### 5.5 Synaptic Dysfunction

Synaptic transmission in dopaminergic neurons involves a tightly regulated sequence of events initiated at the presynaptic terminal. The process begins with the mobilization of synaptic vesicles from reserve pools to the active zone. There, a protein complex composed of syntaxin‑1, SNAP‑25, and synaptobrevin assembles into the SNARE machinery, enabling vesicle docking and priming [191, 192]. Subsequent membrane depolarization triggers the opening of voltage‑gated calcium channels, leading to a localized influx of Ca^2+^, The binding of calcium to synaptotagmin serves as the molecular trigger for the rapid fusion of synaptic vesicles and exocytotic release of dopamine into the synaptic cleft [193]. Released dopamine diffuses across the cleft and activates postsynaptic dopamine receptors to initiate intracellular signaling cascades. To sustain neurotransmission, vesicle membranes are subsequently retrieved through clathrin‐mediated endocytosis and recycled for maintaining neurotransmission [192, 194].

However, in PD, this finely tuned process is disrupted by a cascade of pathological alterations. At the presynaptic level, misfolded α‑syn aggregates accumulate in the terminal, binding to membranes and altering curvature [195]. This disrupts the assembly of the SNARE complex, hindering vesicle docking and reducing dopamine release efficiency [196]. Furthermore, mutations in LRRK2 and VPS35 compromise vesicle trafficking and endosomal recycling pathways, disrupting synaptic vesicle turnover [197, 198, 199]. Calcium‑dependent exocytosis may also be impaired through altered synaptotagmin function or alterations in the lipid composition of synaptic membranes [200]. Beyond presynaptic deficits, chronic dopamine depletion leads to postsynaptic adaptations, including downregulation of D2/3 receptors and inflammation‑mediated desensitization of downstream signaling pathways [201]. Collectively, these synaptic deficits markedly diminish neurotransmission efficacy within the nigrostriatal circuit. Notably, synaptic dysfunction precedes dopaminergic neurodegeneration, highlighting its potential as an early intervention target [195, 202].

### 5.6 Dysregulated Iron Metabolism and Ferroptosis

Iron dyshomeostasis is a well‑established contributor to dopaminergic neurodegeneration in PD. Under physiological conditions, iron supports essential roles in mitochondrial respiration, neurotransmitter synthesis, and myelination. In PD, pathological iron accumulation is consistently observed in the SNpc, as evidenced by R2 MRI relaxometry [203] and postmortem analyses revealing dysregulated expression of iron storage proteins [204]. This iron overload results from iron dysregulation. Increased iron uptake occurs through upregulation of transferrin receptor 1 and divalent metal transporter 1, coupled with impaired iron export and storage due to reduced expression of ferroportin expression and dysfunction ferritin buffering [205, 206]. The expanded labile iron pool enhances Fe^2^⁺‑dependent Fenton reactions, generating excessive ROS and inflicting oxidative damage. Sustained oxidative stress promotes ferroptosis, an iron‑dependent form of programmed cell death characterized by lipid peroxidation and inactivation of GPX4 [207, 208]. In PD models, markers of ferroptosis include decreased GPX4 activity, GSH depletion, and elevated lipid peroxides [208]. Additionally, increased expression of enzymes such as ACSL4 and LPCAT3 promotes the incorporation of polyunsaturated fatty acids (PUFAs) into phospholipids, sensitizing membranes to peroxidation [209, 210]. Importantly, pharmacological inhibition of ferroptosis using ferrostatin‑1 or iron chelation with deferiprone has been shown to protect dopaminergic neurons and ameliorate motor deficits in toxin‑induced PD models [207], highlighting the therapeutic potential of targeting iron‑mediated toxicity.

Beyond its role in ferroptosis, iron overload also facilitates α‐syn misfolding and aggregation. Iron binds to the N‑terminal metal‑binding domain of α‑syn, stabilizing its β‑sheet‑rich conformation and accelerating the formation of oligomerization [211]. Aggregated α‑syn, in turn, impairs intracellular iron homeostasis, creating a self‑reinforcing vicious cycle wherein iron dysregulation and protein aggregation [206]. The convergence of ferroptosis and α‑syn pathology underlines the multifaceted role of dysregulated iron metabolism in exacerbating neurodegeneration in PD.

### 5.7 Gut Microbiome

Alterations in gut microbial composition have emerged as upstream events in the molecular pathogenesis of PD. Characteristic alterations include a reduced abundance of commensal genera such as *Prevotella* and *Faecalibacterium*, and an increase in opportunistic pathogens like *Enterobacteriaceae* and *Proteobacteria* [212]. Through compositional shifts and metabolic alterations, microbial communities may promote α‑syn misfolding, dysregulate immune responses, and increase the production of neurotoxic metabolites [213].

Dysbiotic gut bacteria may trigger α‑syn misfolding and aggregation through inflammatory signals. Within the enteric nervous system, proinflammatory conditions can induce α‑syn overexpression and facilitate oligomer formation. Bacterial products such as LPS, predominantly derived by gram‑negative organisms, can activate microglia TLR4 [214, 215], enhancing α‐syn uptake, oxidative stress, and cytokine production—all contributing to neurotoxicity [216, 217, 218].

Microbiota changes also impact the host immunity and epithelial function through metabolite regulation. A depletion of SCFA‑producing bacteria, including *Prevotella*, *Roseburia*, and *Faecalibacterium prausnitzii*, has been consistently observed in PD [22, 127, 129]. The consequent decline in SCFA levels has been linked to impaired inflammation regulation and reduced epithelial barrier integrity. SCFAs are known to signal through G‑protein‑coupled receptors (such as GPR41 and GPR43) and histone deacetylase inhibition, pathways that play important roles in maintaining mucosal homeostasis [212, 219]. When this regulation is disrupted, proinflammatory mediators produced in the gut environment are more likely to enter systemic circulation and affect distant tissues.

Microbial‑derived metabolites have also been implicated in central disease progression. Elevated production of neurotoxic molecules, such as trimethylamine N‑oxide, secondary bile acids, and tryptophan‑derived compounds, has been linked to altered microbial composition [25, 220]. These metabolites are generated by bacteria such as *Clostridium* and *Desulfovibrio*, which are frequently enriched in PD [25]. After entering the bloodstream, these molecules may interact with brain‑resident immune cells, amplify neuroinflammation, and exacerbate neuronal dysfunction.

Collectively, these mechanisms underscore the role of the gut microbiota as an active modulator of PD pathogenesis. Through microbial‑induced protein misfolding, immune dysregulation, and metabolic toxicity, the gut microbiome contributes to multiple molecular events that converge on neurodegeneration.

### 5.8 Cholesterol Metabolism

Cholesterol in the brain is synthesized primarily by astrocytes and delivered to neurons via apolipoprotein‑mediated transport [221, 222]. Concentrated in neuronal membrane microdomains known as lipid rafts, cholesterol modulates protein distribution and synaptic signaling. Due to limited turnover in the CNS, even modest metabolic dysregulation can lead to local cholesterol accumulation [223]. This accumulation contributes to α‑syn pathology through multiple mechanisms.

One such mechanism involves the direct interaction between α‑syn and cholesterol‐rich membranes. Through its N‑terminal domain, α‑syn preferentially binds to lipid rafts, adopting an oligomerization‑prone α‑helical conformation [221, 224]. In vitro models demonstrate that high cholesterol content markedly accelerates α‑syn aggregation, suggesting that these microdomains act as nucleation sites for LB formation [225, 226].

A second mechanism centers on lysosomal dysfunction. Accumulated cholesterol in late endosomes and lysosomes impairs NPC1‑mediated trafficking and disrupts acidification, thereby inhibiting autophagic flux and reducing α‑syn degradation [227, 228]. This process is further exacerbated by genetic mutations in GBA1, which independently compromise lysosomal enzyme activity and membrane integrity [229, 230]. The synergy between cholesterol overload and *GBA1*‑related lysosomal impairment amplifies α‑syn accumulation [231].

Excess cholesterol also generates oxidized metabolites, such as 24S‑ and 27‑hydroxycholesterol, which cross the BBB and activate TLR4‑dependent pathways in microglia [232]. This triggers NF‑κB‑mediated cytokine production and oxidative stress, compounding α‑syn toxicity and increasing dopaminergic neuron vulnerability [233, 234].

The key molecular mechanisms of PD pathogenesis form a set of interconnected processes that act synergistically to promote α‑syn aggregation and neuronal degeneration. Moreover, these mechanisms provide the provide specific molecular pathways that link cellular pathology with the systemic axes discussed earlier. Clarifying these molecular underpinnings is therefore essential for establishing mechanistic bridges between cellular dysfunction and systemic disease features, and for identifying precise molecular targets for therapeutic intervention.

## 6 Prevention Strategies and Therapeutic Approaches

Preventive and therapeutic strategies in PD reflect a growing emphasis on early intervention and personalized management. This section provides a structured overview of current efforts in both prevention and treatment, beginning with primary and secondary prevention approaches that target modifiable risk factors and early disease processes. It then explores mainstream and emerging therapeutic modalities, including pharmacological, surgical, and microbiota‑based interventions. By examining both established practices and experimental strategies, this section aims to clarify the translational relevance of recent findings and identify key challenges in optimizing care across disease stages.

### 6.1 Prevention Strategies

#### 6.1.1 Primary Prevention: Reducing Toxin Exposure

Primary prevention of PD focuses on mitigating modifiable risk factors to delay or prevent disease onset [235, 236]. Key strategies involve minimizing toxin exposure and adopting lifestyle interventions. Environmental toxins play a crucial role in PD pathogenesis, with substantial evidence linking them to dopaminergic neuron degeneration [236]. Pesticides like rotenone and PQ, along with heavy metals such as lead and manganese, disrupt mitochondrial function and exacerbate oxidative stress—both pivotal mechanisms in PD advancement. A meta‑analysis investigating metal exposure revealed a 2.1‑fold increase in PD risk (OR = 2.10, 95% CI: 1.63–2.71) associated with prolonged manganese and copper exposure [92]. Furthermore, industrial pollutants like PM2.5 are implicated; a multicounty cohort study showed high PM2.5 exposure (>20 µg/m^3^) correlated with a 1.3‑fold rise in PD incidence. Preventative actions involve regulating agricultural pesticide, enforcing safety protocols for metal industry workers, and enhancing urban air quality [237, 238]. Public health policies should prioritize reducing community exposure to these toxins, particularly in regions with high PD prevalence.

Lifestyle factors such as diet, exercise, and smoking significantly influence the risk of PD [92, 235, 236, 237, 238, 239]. The consumption of a Mediterranean diet, known for its high antioxidant content (e.g., polyphenols in fruits, omega‑3 fatty acids in fish), is linked to a 30% lower risk of PD. This dietary pattern mitigates oxidative stress and neuroinflammation, key contributors to dopaminergic cell loss [240]. Conversely, high red meat and saturated fats intake may elevate the risk of PD by fostering systemic inflammation. Engaging in regular physical activities such as aerobic exercise and tai chi can enhance motor function and potentially serve as a protective factor against PD. A prospective cohort study involving 143,325 participants revealed that engaging in moderate‑to‑vigorous exercise (>150 min per week) was associated with a 25% decrease in the risk of PD (HR = 0.75, 95% CI: 0.63–0.89) [241]. Exercise promotes neuroplasticity and boosts the levels of BDNF, which in turn supports the survival of dopaminergic neurons [242, 243].

Mendelian randomization analyses reveal an inverse association between smoking and PD risk (OR = 0.83, 95% CI: 0.72–0.95) [244, 245]. Nevertheless, the detrimental effects of smoking, such as cancer and cardiovascular diseases, significantly outweigh this potential advantage, rendering it an inadvisable preventive measure. Similarly, moderate alcohol consumption (1–2 drinks per week) may reduce the risk as well (OR = 0.76, 95% CI: 0.64–0.91), possibly due to its anti‑inflammatory properties; however, excessive intake counteracts these potential benefits [246].

#### 6.1.2 Secondary Prevention (Early Disease Stages)

Secondary prevention strategies aim to intervene during the early or preclinical stage of PD, particularly in high risk individuals, with a focus on early detection and neuroprotective interventions [236]. Biomarker development is essential for identifying at‑risk populations prior to the onset of overt motor symptoms [235, 236]. Promising biomarkers include α‑syn aggregates in CSF and blood [28]. A meta‑analysis revealed that CSF α‑syn levels were markedly reduced in individuals with PD compared with controls, with seed amplification assays demonstrating a sensitivity of 88% and specificity of 95% [247]. Neuroimaging biomarkers also offer substantial predictive value, for example, reduced DAT uptake on PET scans can detect dopaminergic deficits 5–10 years before clinical symptom onset and help predict conversion to manifest PD [248, 249]. Additional biomarkers like neuroinflammatory markers (e.g., TNF‑α, IL‑6) and genetic variants (e.g., LRRK2, GBA mutations) contribute to early risk stratification [64, 79, 173, 250]. The combination of these biomarkers enhances diagnostic accuracy, enabling timely interventions before irreversible neuron loss occurs.

Several pharmacological agents are under investigation for early therapeutic intervention in PD. GLP‐1 receptor agonists such as liraglutide and exenatide, which are commonly prescribed for type 2 diabetes, have shown potential to decrease α‑syn aggregation and improve mitochondrial function [251, 252, 253, 254, 255]. In a case–control study, exenatide treatment over a 12‑month period led to a 15% improvement in UPDRS scores in early‑stage PD patients, alongside a reduction in CSF neuroinflammatory markers [256]. Iron chelators also represent a promising approach, as excess iron accumulation promotes oxidative stress and contributes to dopaminergic neuron degeneration [207, 257, 258]. A phase II randomized controlled trial demonstrated that deferiprone, an iron chelator, significantly reduced iron accumulation in the substantia nigra accompanied by measurable improvements in motor function assessed by UPDRS [259]. Anti‑inflammatory approaches are also being explored. Nonsteroidal antiinflammatory drugs (NSAIDs) may inhibit microglial activation and thereby slow disease progression [260]. A meta‑analysis supported this notion, indicating that regular NSAID use was associated with a 20% reduction in the rate of PD progression (HR = 0.80, 95% CI: 0.68–0.94) [261].

### 6.2 Therapeutic Approaches

#### 6.2.1 Cross‐Sectional Comparison of Therapies

Therapeutic strategies for PD can be broadly categorized into mainstream and emerging approaches, each differing in mechanisms, efficacy, and limitations (see Table 2). Among established treatments, pharmacological interventions remain central. Levodopa remains the most effective agent for motor symptoms, with randomized trials showing a 30–40% improvement in UPDRS scores [262]. However, long‐term administration frequently leads to motor fluctuations and dyskinesia, affecting approximately 50% of patients within 5 years. Dopamine agonists, including pramipexole, provide comparable efficacy but increase the risk of impulse control disorders [262]. In parallel, rehabilitative therapies such as balance training and speech therapy have shown benefits in both motor and nonmotor domains [263, 264]. A 2022 meta‐analysis demonstrated that structured aerobic exercise led to moderate improvements in motor function (UPDRS‐III), with a standardized mean difference of –0.40 (95% CI: −0.55 to −0.24), and greater benefits observed in high‐compliance programs (SMD = −0.79, 95% CI: −1.44 to −0.13) [265]. For advanced cases, deep brain stimulation targeting the subthalamic nucleus effectively reduces motor fluctuations by up to 60% (36–39), though it carries procedural risks including infection (3–5%) and cognitive adverse effects (10–15%) [266, 267].

**TABLE 2:** Comparison of therapeutic approaches for PD.

Therapeutic approach	Mechanism of action	Efficacy highlights	Key limitations	References
Levodopa/carbidopa	Dopamine precursor converted to dopamine in the brain	Gold standard for motor symptom relief 50–60% improvement in UPDRS scores	Long‑term use causes dyskinesias (30–50% incidence at 5 years) Wearing‐off phenomenon	[277, 278]
Dopamine agonists	Direct D2/D3 receptor stimulation	Delay levodopa initiation by 2–3 years Better tolerability in younger patients	Hallucinations (10–15%) Impulse control disorders (5–8%)	[277, 278]
MAO‑B inhibitors	Inhibit dopamine breakdown in CNS	Reduce “off” time by 1.5–2 h/days Neuroprotective potential in early stages	Modest efficacy (UPDRS improvement: 3–5 points) Serotonin syndrome risk	[277]
COMT inhibitors	Block peripheral dopamine metabolism	Prolong levodopa effect by 30–40% Reduce motor fluctuations	Diarrhea (20–30%) Liver toxicity (tolcapone)	[277, 278]
Deep brain stimulation	High‑frequency stimulation of STN/GPi	60–70% reduction in motor scores 5–7 years symptom control	Surgical risks (5–10% infection) Cognitive decline in 15% patients	[277, 278]
α‑Synuclein‑targeted therapies	Inhibit aggregation/clearance of toxic α‐synuclein	Phase 2 trials show 30% reduction in Lewy body burden	Limited blood–brain barrier penetration Mixed target specificity	[279, 280]
GDNF/GDNF family therapies	Neurotrophic factor delivery to substantia nigra	Preclinical: 40% dopaminergic neuron preservation	Invasive intraparenchymal delivery Variable gene expression efficacy	[280]
Physical therapy	Multimodal sensorimotor retraining	Significant improvement in UPDRS3 score Greater effectiveness in older patients (≥70 years) Improvements in balance (BBS), fall risk (TUG, FTSST), gait (step velocity, preswing angle), muscle strength (knee extension torque), and quality of life (PDQ‑39)	Short intervention duration (4 weeks); long‑term effects not evaluated Relies on therapist availability and proximity	[281]
Gene editing	Knockdown *SNCA* or correct *LRRK2* mutations	Preclinical: 50–70% reduction in α‑synuclein aggregates	Off‐target effects Ethical concerns in germline editing	[279, 280]
Fecal microbiota transplantation	Transplant healthy donor microbiota to restore gut–brain axis balance	2024 RCT: 5.8‐point improvement in MDS‐UPDRS motor scores vs. 2.7 in placebo	Short‑term efficacy (12 weeks); donor–microbiota compatibility issues	[282]
Plasma infusion therapy	Infusion of young plasma/albumin to reduce neuroinflammation and α‑synuclein aggregation	2020 trial: 15% UPDRS‑III improvement; reduced TNF‑α by 30%	Limited sample size (*n* = 15) No long‑term safety data	[283]

Emerging therapies are gaining increasing attention for their potential to modify disease progression. Cell‐based approaches, such as transplanting dopaminergic progenitor cells derived from induced pluripotent stem cells, have shown motor improvements in animal models [268, 269, 270, 271]. A meta‑analysis of 11 clinical trials revealed that allogeneic cell transplantation led to a significant decrease of 16.5 points (95% CI: 8.1–24.9) in “off‐state” UPDRS scores at 12 months [272], with sustained benefits observed for up to 36 months [271]. Nonetheless, challenges remain, including graft‑induced dyskinesia in 10–15% of patients and inconsistent cell survival [269, 272]. Microbiome‑targeted interventions, particularly fecal FMT, have also show therapeutic potential. In a phase I trial involving 56 PD participants, FMT improved constipation, which is a common nonmotor symptom in PD, and exhibited preliminary signals of motor benefit [273].

In comparison, conventional therapies remain effective for symptom control, while emerging strategies aim at disease modification, albeit with varying effectiveness at this early stage (see Table 2 for a detailed comparison). Cell‑based therapies show promise in providing sustained motor amelioration, whereas microbiome interventions target nonmotor symptoms [274, 275]. In contrast, the application of transfusions‑based strategies in PD remains experimental and requires further validation [273, 276].

#### 6.2.2 Transfusion Therapy

Transfusion‐based strategies in PD aim to restore plasma‐derived neurotrophic and anti‐inflammatory factors that decline with aging and disease progression [283]. Preclinical studies, transfusion of young plasma attenuates microglial activation, preserves dopaminergic neurons, and reduces α‐syn accumulation in the substantia nigra [284, 285]. These beneficial effects are associated with elevated levels of insulin‐like growth factor 1 (IGF‐1) and vascular endothelial growth factor (VEGF). IGF‐1 not only supports neuronal survival via the PI3K/Akt pathway but may also reduce α‐syn aggregation [286, 287]. VEGF strengthens the BBB and promotes vascular health, helping to stabilize the neuronal environment [288]. In contrast, older individuals and PD patients have reduced circulating IGF‐1 and an altered IGF‐1/IGFBP ratio, which may weaken systemic neuroprotection [289, 290].

While preclinical studies demonstrated promising effects in animal models of PD, clinical translation remains challenging due to ethical, logistical, and biological constraints. A large cohort study involving over 1.4 million transfusion recipients found no increased PD risk in those who received blood from donors later diagnosed with neurodegenerative diseases, supporting transfusion safety [291]. In a Phase I open‐label trial, repeated infusions of young plasma in 15 PD patients were well tolerated and associated with modest improvements in fatigue and quality‐of‐life measures, but no control group or biomarker tracking was included [283]. Key results, proposed mechanisms, and limitations across relevant animal, observational, and clinical studies are summarized in Table 3. PD‐specific factors further complicate clinical translation. The progressive nature of the disease means that short‐term interventions are unlikely to reverse established pathology. Although candidate biomarkers such as neurofilament light chain, growth hormone‐releasing hormone, aminoacylase 1, α‐syn, and glucosylsphingosine have been proposed, none reliably capture short‐term therapeutic effects due to variability and weak clinical correlation [292, 293, 294, 295].

**TABLE 3:** Blood‐based interventions and their impact on PD and neurodegenerative diseases.

Research type		Disease name	Intervention method	Main findings	Potential mechanistic hypotheses	Limitations	Reference articles
PD treatment research	Animal experiment (mice)	PD	Connected wild‐type and GFP transgenic C57BL/6 J mice via parabiotic surgery; one received α‐synuclein aggregates injection; analyzed serum and brain	Injected mice had neuron loss and increased phosphorylated α‐synuclein. Parabiotic partners showed no such changes.	If blood flow spreads α‐synuclein, transmission should occur. But results did not confirm it, maybe due to experimental factors.	Surgery and injection on same day may miss changes. Plasma α‐synuclein level did not match nonprimate studies. Enteric nervous system transmission not ruled out. Aggregates may have other components and different dosage.	[309]
	Animal experiment (mice)	PD	Conducted heterochronic parabiotic surgery on A53T and wild‐type mice; measured Hu‐α‐syn levels and protein expression	Wild‐type mice had increased Hu‐α‐syn in blood and substantia nigra. A53T mice showed no change.	Exogenous Hu‐α‐syn may cross the blood–brain barrier in wild‐type mice. A53T mice may have autophagy–lysosome pathway impairment.	Antibody for fila‐α‐syn is nonhuman‐specific. WT‐para mice's Hu‐α‐syn entry to brain parenchyma needs verification.	[310]
	Retrospective cohort study (humans)	Neuro‐degenerative diseases (PD)	Tracked patients' disease status using Swedish and Danish databases; analyzed with Cox regression	2.9% received transfusions from donors with neurodegenerative diseases, but no transmission was found.		Observational study may have missed diagnoses. Patient registry may be incomplete. Database cannot be externally verified. Some disease subtypes may not be detected.	[291]
	Clinical trial (open‐label, phase I) (humans)	PD	15 patients received young fresh frozen plasma infusions; assessed motor, cognitive, and other functions	Infusions were safe. Patients improved in phonemic fluency and PDQ‐39 stigma sub‐score. TNF‐α levels decreased.	Young plasma may regulate inflammation, reducing TNF‐α to relieve neuroinflammation.	Small sample size; no multiple testing correction; placebo effect may exist.	[283]
Other neuro‐degenerative disease treatment research	Experimental study (mice)	AD	Established 5XFAD‐B6 parabiotic model; compared TREM2‐deficient and normal 5XFAD mice	Aβ‐reactive myeloid cells came from resident microglia. TREM2 deficiency changed Aβ plaque structure and increased neuronal damage.	TREM2 protects in early AD by promoting microglia function around Aβ plaques.	Results may not apply to humans. TREM2's mechanism is not fully clear.	[311]
	Experimental study (mice)	AD	A parabiotic model was established between APPswe/PS1dE9 transgenic AD mice and their wild‐type littermates; wild‐type mice were intravenously injected with HiLyte Fluor 488‐labeled Aβ42 or _125_I‐labeled hAβ for experiments.	(1) Blood‐derived hAβ could enter the brains of wild‐type mice and accumulate, forming cerebral amyloid angiopathy (CAA) and Aβ plaques. (2) It induced AD‐related pathological changes in the brains of wild‐type mice, such as hyperphosphorylation of tau protein, neurodegeneration, neuroinflammation, and microhemorrhage. (3) It significantly impaired long‐term potentiation (LTP) in the CA1 region of the hippocampus of wild‐type mice.	Aβ in the blood can enter the brain and induce Aβ‐related pathological changes in the brain through a prion‐like mechanism, affecting neuronal function and leading to cognitive dysfunction.	The mouse model used in the experiment is different from human AD and cannot fully simulate the human disease state. The specific molecular mechanism by which blood‐derived Aβ enters the brain and its universality in humans have not been clarified.	[312]
	Case–control study (humans)	Dementia	Forty patients with dementia onset before the age of 70 years were selected as the case group, and 80 community controls matched for age, gender, and race were selected as the control group. Various factors were compared and analyzed between the two groups.	No significant association was found between blood transfusion and AD.	Not mentioned	The sample size was relatively small. Other complex factors affecting AD may not have been fully considered.	[313]
	Case–control study (humans)	AD	In seven centers in Italy, 116 patients clinically diagnosed with AD were selected as the case group. Hospital controls (160 cases) and population controls (97 cases) matched for age, gender, and residence were selected as the control groups respectively. The relationship between blood transfusion and AD was analyzed.	No association was found between blood transfusion and AD.	Not mentioned	The sample size was limited. The study was confined to a specific region, and there may be regional limitations.	[314]
	Case–control study (humans)	AD	170 patients aged 52–96 years with a clinical diagnosis of AD were selected as the case group, and 170 controls matched for age and gender and, as far as possible, for general birth habits were selected as the control group. The association between blood transfusion and AD was evaluated.	No association was found between blood transfusion and AD (OR = 0.63).	Not mentioned	The sample size was relatively small. It was difficult to comprehensively control confounding factors.	[315]
	Case–control study (humans)	AD	252 AD patients who had lived in Olmsted County for at least 40 years were selected as the case group, and 252 controls matched for age and gender were selected as the control group. Whether blood transfusion was a risk factor for AD was determined.	Exposure to at least one blood transfusion, three or more blood transfusions, and at least six blood transfusions were all not associated with an increased risk of AD.	Not mentioned	The sample was limited to the population of a specific region. Some potential confounding factors may have been overlooked.	[316]
	Case–control study (humans)	AD	326 newly diagnosed patients with probable AD were selected as the case group, and 330 controls from the same healthcare facility matched for age and gender were selected as the control group. The relationship between a history of blood transfusion and AD was evaluated.	The frequency of blood transfusion in the control group was higher than that in the case group (crude OR = 0.62). Stratified analysis by APOE‐e4 genotype showed no effect modification, that is, regardless of the APOE‐e4 status, a history of blood transfusion was not associated with an increased risk of AD.	Not mentioned	The sample size may have been insufficient to detect a weak association between blood transfusion and AD. The control of confounding factors may not have been comprehensive enough.	[317]
	Case–control study (humans)	Neurodegenerative diseases	Based on the Scandinavian donation and blood transfusion database, 1,465,845 patients who received blood transfusions between 1968 and 2012 were studied. Patients who received blood from donors diagnosed with neurodegenerative diseases within 20 years were defined as the exposed group, and those who received blood from donors without neurodegenerative diseases were defined as the nonexposed group.	Exposure to blood from affected donors was not associated with the risk of AD (HR = 0.99, 95% CI: 0.85–1.15). No disease consistency was found among multiple recipients of blood from the same donor.	Not mentioned	The study was not specifically focused on AD. There is a delay between the onset and diagnosis of AD, which may lead to an underestimation of the number of recipients exposed to risky blood and the subsequent occurrence of neurodegenerative diseases. The study only focused on the risk to recipients of blood from donors known to have neurodegenerative diseases and did not consider unknown donors or people with no history of blood transfusion.	[291]

To address these challenges, autologous transfusion has been proposed as a strategy to mitigate immunogenic risks and simplify regulatory pathways [296]. However, autologous plasma may lack sufficient levels of key neuroprotective factors due to age‐ or disease‐related declines, potentially limiting its therapeutic efficacy [297]. One possible strategy is to enhance autologous plasma with recombinant neurotrophic factors such as IGF‐1 or BDNF [114, 298]. This combined approach may help balance the safety of autologous transfusion with the therapeutic potency seen in healthy donor plasma. BDNF supports neuronal growth through TrkB signaling but exhibits BBB penetration [299]. In PD mouse models, focused ultrasound has been shown to improve its brain delivery. Another candidate, glial cell line‐derived neurotrophic factor (GDNF), has shown mixed results in early trials and faces similar delivery issues [114, 299]. To overcome these limitations, gene therapy strategies are being explored to improve safety and targeting [300].

Efforts to improve transfusion consistency have focused on donor standardization. Selecting healthy individuals aged 20–40 years without major comorbidities helps minimize variability in plasma composition and supports reproducibility. Such age groups are associated with higher circulating levels of neurotrophic and anti‐inflammatory factors [297]. Processing methods such as plasma fractionation allow for the enrichment of beneficial components‐including exosomes, while removing potentially harmful elements. Nevertheless, transfusions carry inherent risks, including transfusion reactions (occurring in approximately 1–2% of cases) and the potential transmission of infectious agents such as HIV and hepatitis viruses [301]. Although pathogen reduction methods such as solvent–detergent treatment can mitigate some risks, they may also alter or diminish some beneficial plasma components [296]. Exosome‐based therapy, however, faces challenges for clinical translation. Each milliliter of human plasma contains approximately 10^1^⁰ exosomes, making it difficult to achieve therapeutic doses without large‐volume collection or highly efficient concentration strategies [302, 303]. This limitation raises concerns regarding donor burden, scalability, and the feasibility of repeated clinical administration. While preclinical studies suggest exosomes derived from young plasma can inhibit α‐syn propagation and support neuronal health in PD models [304, 305], but challenges related to standardized production, targeted delivery, and immunogenicity remain to be addressed before clinical application.

In summary, transfusion‐based therapeutic strategies represent a biologically plausible approach to modulate PD pathology through the restoration of systemic homeostasis, particularly by counteracting disruptions in neuro–immune–metabolic balance that are implicated in disease progression [114, 306]. Future studies should initially focus on observational cohorts studies to evaluate whether lower circulating levels of factors such as IGF‐1 or VEGF correlated with accelerated clinical progression or greater symptoms severity in PD patients. In parallel, delivery technologies such as recombinant protein formulations, exosome purification and focused ultrasound should be evaluated in animal models [307, 308]. Subsequently, well‐designed early‐phase clinical trials will be essential to assess safety, dosing, and short‐term biomarker changes before broader use can be considered (see Table 3 for supporting evidence across study types).

#### 6.2.3 Microbial Therapies

Building on extensive evidence linking gut dysbiosis to PD pathogenesis, microbial interventions have emerged as potential therapeutic strategies [19, 20, 21, 22, 123]. Altered microbial composition, marked by decreased diversity and increased proinflammatory taxa, has been associated with intestinal barrier dysfunction, neuroinflammation, and α‐syn pathology. These findings suggest that restoring microbial homeostasis may offer a disease‐modifying approach. Accordingly, FMT, probiotics, and other microbial therapies have gained interest for their ability to modulate gut‑brain signaling and improve clinical symptoms.

FMT has shown promise in alleviating gastrointestinal dysfunction in PD, as evidenced by both case studies and controlled trials (see Table 4). In an open‑label study involving 20 patients, constipation scores improved by 30%, although the absence of a control group limits interpretation [22]. Meta‐analyses and clinical trials have shown inconsistent outcomes, with no significant effect of FMT on motor symptoms in PD [318]. Probiotics have demonstrated anti‑inflammatory and promotility symptoms in preclinical studies and small clinical trials [319], but long‐term clinical outcomes remain inconclusive. While dietary modulation may assist in shaping microbial composition, it is typically considered adjunctive rather than a standalone microbial therapy. A detailed summary of animal and clinical studies investigating microbial transplantation in PD is presented in Table 4.

**TABLE 4:** Microbiome transplantation in PD: animal and clinical research.

Research category	Experimental subjects	Experiment type	Experimental method	Key findings	Limitations	References
Animal research	PD mice	Gut microbiota transplantation	Transplanted the gut microbiota of normal mice into PD mice	Reversed gut microbiota imbalance, alleviated gut and neuroinflammation; decreased short‐chain fatty acid levels and increased striatal dopamine and 5‐hydroxytryptamine	Differences exist between the animal model and human PD pathology; the direct mechanism between microbiota regulation and neurodegeneration remains unclear.	[325]
	Adult PD mice	Antibiotic intervention	Administered antibiotic treatment to adult PD mice and germ‐free mice	Antibiotics alleviated PD symptoms; short‐chain fatty acids (acetate, propionate, and butyrate) exacerbated motor disorders and neuroinflammation.	Germ‐free mice lack normal microbiota, making it difficult to directly extrapolate the results to the general population; long‐term effects were not explored.	[22]
	Rotenone‐induced PD mice	Antibiotics + FMT	Established a rotenone‐induced PD model, followed by antibiotic treatment and fecal microbiota transplantation (FMT)	Rotenone caused gut microbiota imbalance and alpha‐synuclein aggregation; FMT improved motor disorders, gut inflammation, and blood–brain barrier damage.	The model does not fully mimic the complex pathology of human PD; the key microbiota components responsible for the effectiveness of FMT remain undefined.	[326]
Clinical research	A 71‐year‐old PD patient	Single‐case FMT	Transplanted the gut microbiota of a 26‐year‐old healthy individual into a 71‐year‐old PD patient	Lower limb tremors were significantly reduced and constipation was alleviated; beneficial bacteria increased while harmful bacteria decreased. Anxiety, depression, and sleep disorders improved.	Extremely small sample size (only one case); lack of a control group; long‐term efficacy and safety were not evaluated.	[327]
	11 PD patients with constipation	FMT	Conducted FMT on PD patients with constipation	Motor symptoms (tremors and rigidity) were reduced. Gut microbiota diversity was restored.	Small sample size; no control group; the causal relationship between microbiota changes and symptom improvement was not clarified.	[328]
	22 PD patients with severe constipation	FMT	Performed FMT on PD patients with severe constipation	Constipation was continuously and significantly improved, and the quality of life was enhanced.	Lack of long‐term follow‐up (only 12 weeks); motor symptom changes were not evaluated; no placebo control group was set.	[329]
Clinical trial	46 early‐stage PD patients	Double‐blind placebo–controlled	Early‐stage PD patients were randomly assigned to receive either healthy donor FMT or autologous stool placebo and were followed up for 12 months.	The FMT group showed a significant improvement in the MDS‐UPDRS motor score (+5.8 vs. +2.7 points).	Moderate sample size (*n* = 46); incomplete blinding; the specific mechanism by which FMT improves motor symptoms remains unclear.	[282]

The translation of these microbial inventions into clinical practice faces several operational challenges. High individual variability in microbiome composition limits the standardization of treatment effects, FMT currently lacks unified protocols for donor screening, fecal material processing, and administration [274, 275, 282, 320]. Regulatory frameworks also varies geographically, with ongoing debate as to whether FMT should be regulated as a biological product or drug. Probiotic formulations often require cold‑chain storage and daily dosing, raising logistical burdens for consistent clinical use.

Microbial therapies also face intrinsic biologicaconstraints. Donor–recipient mismatch in FMT and poor colonization of many probiotic strains limit durable effect [274, 275, 321]. Future directions may involve the development of engineered probiotics with improved tissue targeting, personalized FMT protocols based on metagenomic and metabolomic profiling, and applying machine learning to predict individual responses. PD‐specific microbial markers such as elevated *Escherichia*/*Shigella* linked to motor severity may help to guide targeted interventions [123, 320]. Emerging alternatives, including sterile fecal filtrates and bacteriophage‐based therapies may offer reduced infection risks and greater specificity, although clinical validation remains necessary [322, 323].

Safety remains paramount. FMT is generally well tolerated, but rare cases of *Clostridioides difficile* infection have been reported in PD patients, particularly those with pre‐existing dysbiosis or prior antibiotic exposure [282, 324]. Rigorous donor screening and long‐term postintervention monitoring are therefore essential.

Although microbial therapies are not yet established as frontline treatments, they hold promise as adjuncts approaches for managing non‑motor symptoms and possibly influence disease progression. Their future clinical integration depends on robust randomized trials, regulatory standardization, precision‐guided designs, and mechanistic clarity.

While distinct in their approaches, preventative strategies and therapeutic interventions are intrinsically linked in the management of neurodegenerative diseases. Primary prevention focuses on mitigating risk factors at the population level to reduce disease incidence, whereas secondary prevention aims to intervene in early disease stages to slow pathological progression. A comparative analysis of established and emerging therapeutic modalities‐including transfusion‐based and microbiota‐targeted therapies reveals considerable potential alongside persistent challenges related to ethical considerations, standardized protocols, and mechanistic depth. Moving forward, research should prioritize the integration of insights across preventative and therapeutic domains, fostering synergistic connections between diverse strategies to address these diseases with enhanced precision and efficacy, ultimately leading to improved diagnostic accuracy, treatment experiences, and sustained benefits for patients.

## 7 Conclusions and Prospects

PD poses an increasing global health challenge; epidemiological evidence reveals clear age‐dependent trends, a slightly higher prevalence in males, and striking geographic disparities, particularly between high‐income and resource‐limited regions. These patterns reflect both improved diagnostic capabilities and persistent inequities in access to care and data representation.

Pathogenetically, PD emerges as a multisystem disorder, with peripheral‐to‐central pathways playing critical roles in disease onset and progression. This review highlights three critical pathways, the gut–brain, erythrocyte–brain, and kidney–brain axes as significant contributors to systemic dysfunction. These axes influence core pathogenic mechanisms including neuroinflammation, oxidative stress, metabolic dysregulation, and α‐syn aggregation. Collectively, they offer a cross‐level perspective that links molecular disruptions with clinical manifestations. Therapeutically, traditional dopaminergic treatments remain effective for motor symptoms but offer limited long–term disease control. In contrast, novel strategies grounded in mechanistic insight, which include microbiome modulation, blood‐based interventions, and regenerative cell therapies, stand as emerging directions for disease modification. Preventive approaches such as exercise and Mediterranean dietary patterns also show potential for reducing risk.

Despite these advances, several challenges limit the translation of mechanistic insights into clinical benefits. Molecular processes such as erythrocyte‐related oxidative stress and iron dyshomeostasis vary across individuals, complicating therapy standardization. Systemic contributors including the gut–brain and kidney–brain axes interact with molecular dysfunctions in population‐specific ways. Microbiome therapies targeting gut–brain disruptions face issues with host specificity and response consistency. Blood‐based interventions grounded in erythrocyte–brain mechanisms are constrained by donor variability and logistical feasibility. Cell‐based therapies show promise, yet safety, graft survival, and long‐term efficacy remain concerns.

Epidemiological disparities further complicate progress. Limited diagnostic capacity and biomarker accessibility in low‐ and middle‐income regions restrict early detection and the generalizability of findings on peripheral–central interactions. The epidemiological synthesis relies mainly on data from high‐income settings, potentially underrepresenting region‐specific patterns of risk and response. Moreover, the epidemiological synthesis relies heavily on data from highincome countries, potentially underestimating regional variation in both pathogenic mechanisms and treatment applicability.

Future research should prioritize in‐depth investigation of the gut–brain, erythrocyte–brain, and kidney–brain axes. Clarifying how these systemic pathways interact with molecular dysfunction will be key to understanding disease heterogeneity. Refinement of emerging therapies, including microbiome–based treatments, transfusion strategies, and cell–based regenerative techniques, will require careful consideration of delivery methods, patient‐specific variables, and long‐term safety. Developing personalized interventions also requires a better understanding of how patients respond differently to treatments, all based on a precise understanding of disease mechanisms. Achieving this level of personalization depends on broader data inclusion across diverse populations. Stronger global collaboration is essential to integrate underrepresented regions and to capture the full diversity of PD manifestations and treatment responses. A globally informed yet locally adaptable framework will enable more equitable and effective interventions, ultimately aligning biological insight with clinical utility to improve outcomes for individuals living with PD.

## Author Contributions

Xue‐Yao Guo, Ming‐Yang Wu, and Jing‐Qi Zhang wrote the paper. Xue‐Yao Guo and Ming‐Yang Wu developed the figures and tables. Lin Yuan, Dong‐Yan Song, and Jia‐Yi Li conceived and edited the manuscript. All authors read and approved the final manuscript.

## Funding

This review is supported by the National Natural Science Foundation of China (82201594). Acknowledgements are also to the support of the Swedish Research Council (2023–02216), the Strong Research Environment MultiPark (Multidisciplinary research on Parkinson's disease), Parkinsonfonden (1494/2023), the Innovation and Entrepreneurship Training Program for China Medical University Students (No. 202510159004), and The Brain Foundation (FO2023–0397, FO2025–0296).

## Ethics Statement

The authors have nothing to report.

## Conflicts of Interest

The authors declare no conflicts of interest.

## Data Availability

The data used for epidemiological figures and analysis were derived from the Global Burden of Disease Study 2023, which are publicly available at http://ghdx.healthdata.org/gbd‐2023.
